# Folk knowledge of invertebrates in Central Europe - folk taxonomy, nomenclature, medicinal and other uses, folklore, and nature conservation

**DOI:** 10.1186/s13002-016-0118-7

**Published:** 2016-10-11

**Authors:** Viktor Ulicsni, Ingvar Svanberg, Zsolt Molnár

**Affiliations:** 1 Department of Ecology, Faculty of Science and Informatics of the University of Szeged, Közép fasor 52, 6726 Szeged, Hungary; 2 Uppsala Centre for Russian and Eurasian Studies, Uppsala Univerisity, Box 514, 751 20 Uppsala, Sweden; 3 MTA Centre for Ecological Research, Institute of Ecology and Botany, 2163 Vácrátót, Hungary

**Keywords:** Ethnozoology, Europe, Invertebrate fauna, Ethnomedicine, Nature protection, Edible insects

## Abstract

**Background:**

There is scarce information about European folk knowledge of wild invertebrate fauna. We have documented such folk knowledge in three regions, in Romania, Slovakia and Croatia. We provide a list of folk taxa, and discuss folk biological classification and nomenclature, salient features, uses, related proverbs and sayings, and conservation.

**Methods:**

We collected data among Hungarian-speaking people practising small-scale, traditional agriculture. We studied “all” invertebrate species (species groups) potentially occurring in the vicinity of the settlements. We used photos, held semi-structured interviews, and conducted picture sorting.

**Results:**

We documented 208 invertebrate folk taxa. Many species were known which have, to our knowledge, no economic significance. 36 % of the species were known to at least half of the informants. Knowledge reliability was high, although informants were sometimes prone to exaggeration. 93 % of folk taxa had their own individual names, and 90 % of the taxa were embedded in the folk taxonomy.

Twenty four species were of direct use to humans (4 medicinal, 5 consumed, 11 as bait, 2 as playthings). Completely new was the discovery that the honey stomachs of black-coloured carpenter bees (*Xylocopa violacea*, *X. valga*) were consumed. 30 taxa were associated with a proverb or used for weather forecasting, or predicting harvests. Conscious ideas about conserving invertebrates only occurred with a few taxa, but informants would generally refrain from harming firebugs (*Pyrrhocoris apterus*), field crickets (*Gryllus campestris*) and most butterflies. We did not find any mythical creatures among invertebrate folk taxa. Almost every invertebrate species was regarded as basically harmful. Where possible, they were destroyed or at least regarded as worth eradicating. However, we could find no evidence to suggest any invertebrate species had suffered population loss as a result of conscious destruction. Sometimes knowledge pertaining to the taxa could have more general relevance, and be regarded as folk wisdom concerning the functioning of nature as a whole.

**Conclusions:**

The high number of known invertebrate folk taxa suggests that it would be worth conducting further investigations in other areas of Europe.

## Background

Traditional knowledge systems about the landscape and the biota have been fundamental for human development since the times of pre-modern and pre-industrial societies in Europe. Humans living in close contact with the landscape as herdsmen and peasants have long possessed unified, systematic knowledge, including folk taxonomies, about phenomena that were of importance to them. The use and management of natural resources was based on centuries-old, often millennia-old ecological experience, on multi-generational knowledge passed down from generation to generation [1, 2].

Ethnozoology is the scientific study of the dynamic relationships among people, and animals. Traditional ethnozoological knowledge has great cultural and economical importance. It is widely studied in the tropics and North America (e.g. [3–5]), but also in Europe (e.g. [6, 7]). Wild animal-based natural resources are often among the key resources local communities depend on [8, 9]. A major goal of these communities is to use and manage these resources sustainably (e.g. taboos: [10]; social rules: [11, 12]). Long-term sustainability in the use and management of natural resources requires healthy ecosystems, while at the same time, sustainable management often contributes to maintaining the health of ecosystems [13, 14].

The knowledge passed by local traditional communities, however, not only serves sustainable use and maintenance of the local community and its environment but may also provide valuable data, information and knowledge to science and conservation. Among the potential benefits of traditional ecological knowledge, it can help science to recognize new species (e.g. [15]), provide data on population sizes and dynamics of species that are difficult to observe [16, 17], support the monitoring of ecosystem health, incl. pasture conditions [18, 19], and develop efficient conservation managament strategies and practices [20–23].

There is no reason to imagine that European peasant and herder communities differ fundamentally from native societies in other parts of the world with regard to their ecological knowledge [24]. However, there is scarce information about European folk knowledge of wild invertebrate fauna, including their use in healing and nutrition. Researchers in ethnobiology seldom pay attention to invertebrates in the European context [25]. By contrast, several comprehensive studies have been conducted in other parts of the world. As early as 1887, Stearns published an ethnoconchological work on the use of shells as money among aboriginals of North America [26]. This was actually the first time the prefix “ethno-” was combined with a research field, thus preceding Harshberger’s term “ethnobotany”, coined in 1895 [27]. Another pioneering study was Henderson’s and Harrrington’s ethnozoology of the Tewa people in New Mexico. This study gives a full list of animals, including invertebrates, by order and gives their Tewa names as well as their scientific names [28]. In a comprehensive study Bodenheimer [29] reviewed the ethnographical literature of the use of insects as food worldwide. Nowadays there are several important studies available dealing with ethnobiological aspects of invertebrates. We can, for instance, mention Bentley and Rodríguez [30] on the entire invertebrate fauna of Honduras, and Krause et al. [31] on the insect fauna knowledge of the Roviana people (Solomon Islands). Gurung [32] detailed the knowledge of arthropods among Tharu farmers in Nepal, while Hemp [33] described what the peoples living near Mount Kilimanjaro (Tanzania) knew about invertebrates. A particularly impressive ethnozoological study is Morris [34], dealing with the impact of insects and their classification in Malawi folk culture. In addition, the literature on aquatic and coastal-marine invertebrates is particularly rich (e.g. [35–37]).

The general experience is that many invertebrate species have specific and relevant benefits or detriments, although the number of locally known folk taxa is higher than this [31]. Some culturally salient invertebrate species may even be important keystone species in the lives of certain communities. The majority of these are coastal-marine invertebrates (e.g. shellfish in British Columbia - [37]; crabs (*Ucides cordatus*) in Brazil - [38, 39]). There are fewer culturally salient species among terrestrial invertebrates, and relatively few species have known folk uses (cf. [32, 40]). Keystone species include, among spiders for example, the bird-eating spiders for Afro-Brazilians in Bahia [41], while among lepidopterans there is the Brahmaeid moth on Taiwan [42].

European folk knowledge about invertebrates has, since the nineteenth century, been researched mostly by folklorists and linguists. In 1879–80 the Swedish author Strindberg used a questionnaire to gather valuable data regarding folk names and rhymes connected with the ladybird. His research, using mapping as a method, is a pioneering work in folklore about animals [43]. An encyclopedia was published about Romanian insect folklore, including local names, legends, fables and myths, the role of insects in witchcraft, and beliefs about insects as pests or as omens [44]. Herman published the local names of insects and invertebrate pest species known by Hungarian herders [45]. We can also mention an interesting article on folk knowledge about botflies (Oestridae) found as parasites on domesticated reindeer, published by the ethnographer and linguist Wiklund [46]. This kind of ethnographic folklore-linguistic research tradition continues today in Europe. Wiggen, for instance, inspired by current ethnobiologists, has recently published an exciting study on the traditional names of lower animals in Norway [47]. In European cultures, it is generally quite uncommon to use or consume invertebrates [48, 49]. The only invertebrates with any significant ethnobiological literature are for the taxa of snails [50], slugs [51], leeches [52], ladybirds [6], crustaceans [53], oil beetles [54] and head lice [55], but none of these are cultural keystone species. Here we should also mention a small but intriguing study on Sami children’s knowledge and use of small invertebrates for amusement and to play with [56]. In 2006, Svanberg [57] published a small book with ethnozoological studies on the human relationship with bumblebees, earthworms, froghoppers, isopods, liver flukes, moonjellies and starfish in Scandinavia and Estonia. There is of course extensive biological literature on pests, but very little detailed documentation of folk knowledge has yet been carried out in Europe [58, 59]. We are, however, of the opinion that further data may exist in local languages, in works on ethnography, local history and perhaps even linguistics, but these have not yet entered the international ethnobiological literature (e.g. [60]).

There is also very little Hungarian literature on folk knowledge of invertebrates. Linguistic (dialectic), ethnographic and ethnobiological literature is available concerning 161 invertebrate species in the Sóvidék region in Transylvania [61], 67 taxa along the Danube [62], the beetle taxa *Melolontha melolontha, Lucanus cervus* and *Lytta vesicatoria* [63], and the snail species *Helix* spp. [64]. Sporadic data may also appear in ethnographic and linguistic literature written in the Hungarian language, for example in monographs on farming and forest ethnography, e.g. in Hegyi [65] on *Lytta vesicatoria* and *Melolantha melolantha*. To date, nothing has been published in English about the folk knowledge of invertebrates of the Carpathian Basin.

Our article has the objective of presenting the Hungarian folk knowledge of invertebrate species uncovered in three areas of the Carpathian Basin (in Romania, Slovakia, and Croatia), including:

1. a list of folk taxa of invertebrates,
2. their folk biological classifications and nomenclatures,
3. their salient features, and
4. their uses, related proverbs and sayings, and their conservation.

This is the first article in Europe to deal comprehensively with an entire invertebrate fauna. The folk knowledge, nomenclature and uses of 208 taxa are presented in detail. The high number of known folk taxa suggests that it would be worth conducting further investigations in other areas of Europe.

## Methods

### Study areas

**Fig. 1:**
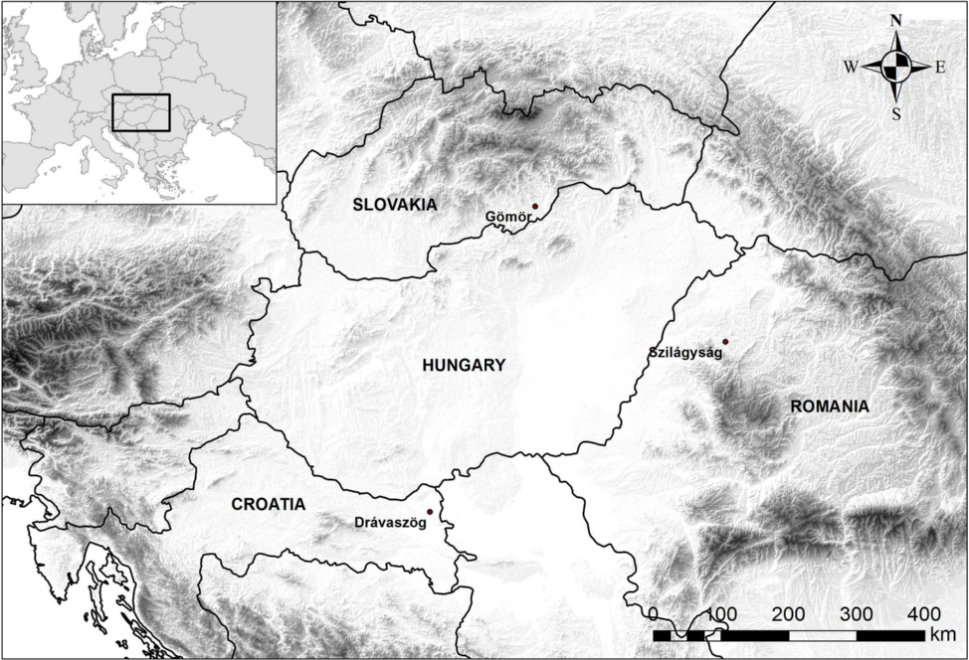
The study areas in Central Europe

We collected data among ethnic Hungarians practising small-scale, traditional agriculture. Our research was conducted in Romania (Sălaj county [Szilágyság], Nușfalău [Szilágynagyfalu] commune), Slovakia (Gemer [Gömör] region, primarily in the municipalities of Vyšné Valice [Felsővály] and Gemerské Michalovce [Gömörmihályfalva]), and Croatia (Baranja region [Drávaszög], mainly around the villages of Lug [Laskó], Vardarac [Várdaróc] and Kopačevo [Kopács] (Fig. 1)). As the people we studied spend a lot of time in the fields and forests during their everyday activities, they still have a close, direct connection to their natural environment. The settlements where the data were collected, each with between 100 and 2500 inhabitants, are characterised by a large amount of abandoned agricultural land, and by ageing populations.

The three study areas are characterised by a moderate continental climate, with a mean annual precipitation of 600–700 mm. The mean annual temperature in the two northern areas is 8–8.5 °C (July mean 19 °C, January mean −4 °C), while in Baranja, further south, it is slightly higher, around 10 °C (July mean 21 °C, January mean −4 °C) [66]. The elevation is 75–90 m.a.s.l. in Baranja, 200–350 m.a.s.l. in Sălaj, and 190–500 m.a.s.l. in Gemer. Gemer and Sălaj typically have closed broadleaved forests (oak), while in Baranja there is a mixture of riparian vegetation, marshland and mixed hardwood gallery forests (oak, ash and elm).

### Data collection and analysis

Data was collected in Sălaj in summer 2010, and in Baranja and Gemer in summer 2012. In each area, the objective was to identify and interview local people with the most extensive knowledge. We employed a number of techniques: in Sălaj we first consulted the local Calvinist priest, and then followed the snowball method; in Gemer we also followed the snowball method, but this time starting with the best informants from earlier ethnobotanical researches; in Baranja we collaborated with the local nature conservation warden, István Tórizs, to meet the people who, in the warden’s view, had the greatest traditional folk knowledge. In total we interviewed 58 people. The overall average age of the interviewees was 75 years (within a range from 36 to 90 years), and the regional average ages were 78 in Sălaj, 74 in Baranja, and 71 in Gemer. All the informants retained memories of traditional forest use and smallholder farming, and some were still practitioners. 55 of the interviewees were Calvinist.

We conducted indoor interviews recorded on a dictaphone (approximately 88 h of recording), since the presentation of living specimens and direct observation of animals in the wild would have been greatly inconvenient for most of the informants. Prior informed consent was obtained before all the interviews, and ethical guidelines suggested by the International Society of Ethnobiology were followed. We studied “all” invertebrate species or species groups potentially occurring in the vicinity of the settlements under investigation. We placed an average of 12 photos of species of similar habitat and size on a sheet of A4 paper, to give interviewees a sense of the context and relative size of each taxon. In many instances during our preliminary study, the differing scale of the pictures had greatly inhibited recognition. Where ambiguous descriptions occurred, further enquiries of the characteristics of the species in question were made in order to facilitate identification of the animal at the finest possible taxonomic level. Detailed lists of invertebrate taxa documented by zoologists were available for the regions studied or for ecologically similar neighbouring regions (e.g. [67–70]). We also included a few species that do not occur in the areas under investigation, in order to check the authenticity of local folk knowledge.

In total we collected 3465 individual data records on 208 folk generics and specifics. We also conducted semi-structured interviews with the majority of informants and carried out picture sorting, during which they were asked to group species according to their own systems. We used these results to reconstruct the folk taxonomy. Figures depicting taxonomic relations were prepared following the method used by Berlin [71]. Circles drawn in solid lines on these figures indicate scientific taxa (one species, one genus, one order, one family), whereas those drawn in small and large dashes represent, respectively, folk taxa and more inclusive folk categories. When circles of scientific taxa overlap, this indicates that certain scientific taxa were viewed as alike (e.g. “it is a house mouse, but of a different kind”). Inclusive categories were established on the basis of data collected by pile sorting, co-references and direct questions. However, it was not our intention to arrange individual taxa according to Berlin’s system of taxonomic levels, since the communities we examined are too heterogeneous for this. For each of the taxa, where possible, we documented the local name (or names), their salient features, their uses, any damage they cause, any personal attitudes expressed towards the taxa (positive, negative or neutral), and related folklore issues. The habitats of the species (see Appendix) were determined based on the interviews, on our own experiences and on the scientific literature.

We have listed our data in tables, and summarised the results broken down according to informant and taxon. We have not carried out a quantitative comparison of the knowledge among the three communities, for the data sets have, in many cases, low sample sizes. The differences between the three areas which are important from a qualitative aspect are presented in the chapter on results and discussion. Literal quotations are in italics, and comments by individual interviewees are separated by a slash.

## Results and discussion

### Folk taxa and unknown taxa

**Fig. 2:**
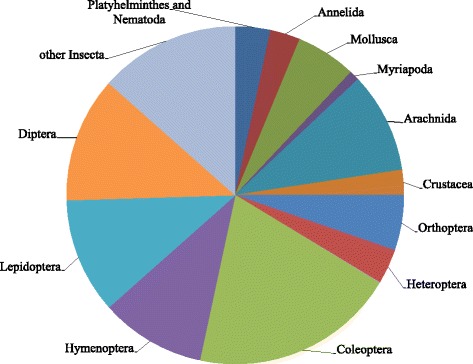
The taxonomic structure of the 208 Hungarian folk taxa of invertebrates documented among ethnic Hungarians in Sălaj, Gemer and Baranja

The folk knowledge of invertebrates revealed in the areas under investigation was extensive and detailed. Folk generics and specifics were documented for a total of 208 invertebrate folk taxa. The majority of these were Coleoptera, Diptera, Lepidoptera, Arachnida and Hymenoptera, while Myriapoda, Crustacea and Annelida were represented with fewer folk taxa (Fig. 2).

Of the 208 folk taxa, in 135 cases (65 %) they could be identified with one or two scientific species, in 28 cases (13 %) with several (3–6) scientific species, and in 45 cases (22 %) with many (more than 6) scientific species.

**Fig. 3:**
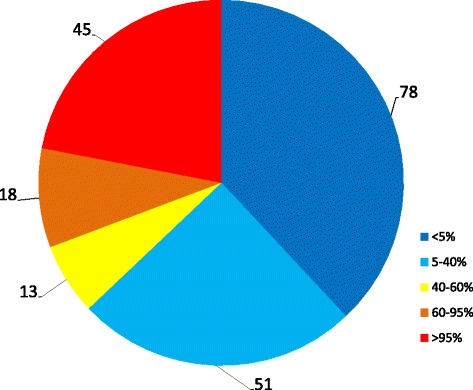
Proportions of species known by different numbers of informants: known by very few (1–3) informant(s) (<5 %); known by 3–23 informants (5–40 %); known by 24–35 informants (40–60 %); known by 36–55 informants (60–95 %); known by “everybody” (56–58 informants) (>95 %)

Certain species were exceptionally well known, but 37.5 % of the taxa were familiar only to between 1 and 3 people (Fig. 3). With certain species or groups of species, the only informants who knew them were those most likely to encounter them because of their profession or as a result of some special activity (such as fishermen using animals as bait, or herders with livestock parasites). 45 taxa (22 %) were known to almost all the informants.

The 208 folk generics and specifics found is greater than the number of known vertebrate folk taxa ([72], Ulicsni ined). Compared with four studies that covered entire faunas [28, 30, 33, 73], the ratio of invertebrate to vertebrate taxa in our region was significant (54 % of specific level taxa). Apart from a single exception (bivalves-frogs, see below), the invertebrates were classified into separate supraspecific groups from the vertebrates, although invertebrates did not constitute a unified group, i.e., an inclusive folk taxon with clearly defined boundaries. This distinction is much sharper, for example, among Mongolians [74].

The differences in the fauna of the three different areas seemed to have little effect on the list of local folk taxa. The faunas of the three areas are similar, as they all contain mostly common, generalist species. The proportion of folk taxa that were restricted to just one of the three areas amounted to only 3.8 % (8 species). For this reason, our analyses treated all the data uniformly. Based on our data, the folk taxonomies could also be regarded – with negligible differences – as uniform (major differences are shown in the results and discussed below).

The distribution of knowledge was not even. Only 36 % of the species were known to at least half of the informants. There are two possible reasons for this: 1) the erosion of knowledge (e.g. reductions in hand harvesting mean less familiarity with the dwarf velvet mite *Microtrombidium pusillum*); 2) certain species are linked to particular farming activities, and so are not generally known. A beekeeper, for instance, would have better knowledge of bee pests, a herder would be more familiar with sheep parasites. Such species may be completely unknown to other members of the community.

The reliability of the knowledge was very high. Despite carrying out constant checks using cross-questioning, errors, falsifications and slips of the tongue were only registered in very few cases. It was more likely for respondents to answer that they didn’t know information or weren’t familiar with species. Due to the general aversion towards the majority of invertebrate species, however, the informants were sometimes prone to exaggeration. A similarly high degree of reliability and low proportion of errors have been experienced in other Central European locations in studies of botanical knowledge [75, 76]. For some species (e.g. vine louse, itch mite), there was a high proportion of knowledge that was not based on personal experience.

In line with our expectations (*cf*. [77, 78]), larger species, those occurring more frequently and those with more distinctive morphologies were more widely known. There was also a greater degree of knowledge of species living in habitats closest to the homes of the informants. Animal and human parasites were often exceedingly well-known. Compared with knowledge of vertebrates, the majority of invertebrate taxa were less detailed. At the same time, a quarter of invertebrate taxa were known to an extent which was comparable to that of the best known vertebrate species.

It was surprising to us that so many invertebrate species are known which have, to our knowledge, no economic significance. The reasons for this were not always clear. Human lifestyles have greatly changed, so there is uncertainty concerning how important a given taxon may have been in the past (e.g. the dormouse species’, which were once regularly hunted, but which are not used at all today, [72]. Yet there were other species that we did not expect to be widely known which proved, during the study, to be significant even today. Examples are species that have appeared recently, such as *Harmonia axyridis*, and species of predatory mites that are particularly small, harmless and can be seen on other insects.

Sometimes only the larval form of an animal is known, such as those of the click beetles (*Agriotes* spp.). In such cases, their place in the taxonomy was less consistent, and often haphazard. The same phenomenon was also often observed in the Sóvidék region (Romania) by Gub [61].

Also surprisingly, informants made no distinction between a significant number of diverse and morphologically easy-to-distinguish lepidopteran species. The hummingbird hawk-moth (*Macroglossum stellatarum*), with its remarkably unique behaviour, was a relatively frequently seen species. Despite being widely known, astonishingly, it was only given a name in one case, and even this was just the name used within the informant’s family.

By comparison, in places where use is made of lepidopteran species (e.g. larvae are eaten in Mexico), up to 67 different species may be known in detail [79]. Species of the order Lepidoptera are an important food source in numerous other regions of the world [80].

We did not find any mythical creatures among invertebrate folk taxa, whereas ethnic Hungarians identify several such animal taxa among vertebrates (e.g. house snake, whistling snake), which are still considered living mythical creatures in the areas under investigation.

With recently settled invasive species or major local invasions of species with a constant lower-level presence, we found that the media played an enhanced role as a source of information. The degree of knowledge sometimes varied greatly, depending on the extent of the invasion, which resulted in some significant differences between the three areas. However, there were only a few species which were known to a varying degree in the three areas (such as the Italian tree cricket, which was more familiar in areas practising viticulture, and *Simulium* spp., in areas where there had previously been major invasions).

### Names – main features and points of interest, unnamed species, modern names

Ninety-three percent of folk taxa had their own individual folk names. The proportion of covert categories was low compared to their higher prevalence among, for example, the Matses of Peru [81]. Where the covert categories are concerned, there is a chance that a few further known folk taxa were not identified during our data collection. The descriptive names used in the case of folk specifics most frequently referred to their morphology or their habitat. A few taxa were only named with the name of the inclusive category.

With some of the taxa, the names given to them within the same community were not consistent. Names could be chopped and changed around even in the case of species that were otherwise clearly separated, such as with locusts, grasshoppers and cicadas; all three of these taxa share the ability to jump, but their size and morphology differ. Almost everybody could distinguish between the three taxa, but the names they used were sometimes swapped around. Berlin et al. [82] also found that people agreed closely on the appropriate names for some species and disagreed markedly on the names of other species.

In a few cases, two or more taxa were given an identical name, even though the fact of their separateness as taxa was widely recognised (e.g. *Lampyris noctiluca*, *Lamprohiza splendidula* and *Cetonia aurata*). The first two are glow worms that light up at night, while the third is a bug (rose chafer) that shines beautifully in sunlight. In our experience, if it was necessary to make a distinction between the first two and the third species, then more knowledgeable informants would, in every case, separate them by adding epithets to the name (e.g. *nappali szentjánosbogár* [daytime Saint John’s bug], or: “the one, which is just a *féreg*”). In everyday speech, however, the context would determine whether the folk specific referred to the first two or to the third species, so there was no need for separate names.

On other occasions, the same folk name was used for completely unrelated and well distinguished taxonomic groups (e.g. *bolha* [flea]: *Pulex irritans* - *Chaetocnema* spp.; *giliszta* [worm]: *Lumbricus* spp. – e.g. *Taenia solium*). The names of folk specifics typically made reference to morphological, habitat and ecological properties. There were also instances of the usefulness of the creature being referred to in its name (*jópióka* – *lópióka,* ‘good leech – horse leech’, *Hirudo medicinalis* – *Haemopis sanguisuga*). Larval forms were given separate names in several instances (e.g. *Hypoderma bovis*, *Melolontha melolontha*, *Pediculus humanus capitis*), even if the larva and the imago comprised the same folk taxon.

There were several taxa with multiple names. The firebug (*Pyrrhocoris apterus*) is a generally known species not only in the areas of our investigation, but generally in regions where Hungarian is spoken [61, 62]. The reason for this may be its distinctive behaviour, or perhaps the fact that hordes of them together can be witnessed in early spring (this phenomenon often also serves as the basis for folk weather forecasts). This species was given a wide range of diverse names. This contradicts the earlier observation [77] that smaller species which cause little or no harm, and which also have no benefit, are often not given names, regardless of how common they are. The proliferation of names also contradicts the observations of Fleck et al. [81], which state, roughly, that the more salient a species is, the more uniform its name will be.

There were far fewer instances of modern names or names used by only one family or individual. Some of these names were humorous, such as *pizsamás bogár* [pyjama beetle] for *Leptinotarsa decemlineata*, or *vízibizigli* (paddled boat) for the waterstriders. This phenomenon has been observed, although similarly infrequently, in botanical studies [83].

Names and other types of knowledge could, in certain cases, be a hybrid of traditional and scientific knowledge. However, the overwhelming majority of the knowledge recorded in our study had a traditional, folk background. Only rarely did some names come to light which derived from formal education or from the media (e.g. *aranyos virágbogár* [golden flower bug] *- Cetonia aurata*; *aranyszemű fátyolka* [gold-eyed veil] *- Chrysopa perla*). It is more common for the official Hungarian scientific names to originate from folk names. The balance in favour of traditional knowledge is stronger for invertebrates than it is for vertebrates [72]. The influence of schooling could only be felt among a few informants and only for a very limited number of species. In Appendix, all the names used by local people which demonstrably originate from “modern” sources (school, media, books, etc.) have been underlined.

### Folk taxonomy, folk nomenclature and salient features

**Fig. 4:**
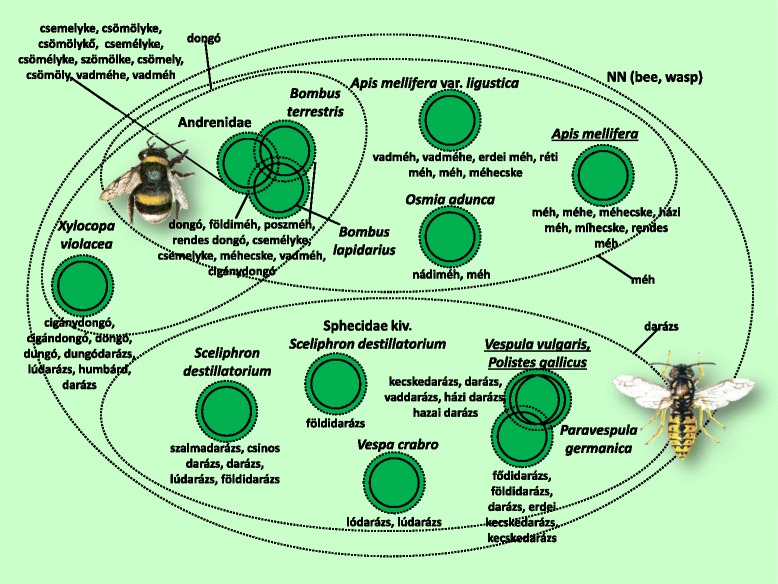
Folk taxa containing bees (Apidae), as well as mining bees (Andrenidae), wasp species (Vespidae) and family of parasitoidal wasps (Sphecidae). Continuous circles on these figures indicate scientific taxa (one species, one genus, one ordo, one family), whereas small and large dashed circles represent folk taxa and more inclusive folk categories, respectively. The overlap of the circles of scientific taxa indicates that certain scientific taxa are viewed as ’alike’. Prototypic species are marked with undelining. NN means the inclusive taxon is not named

**Fig. 5:**
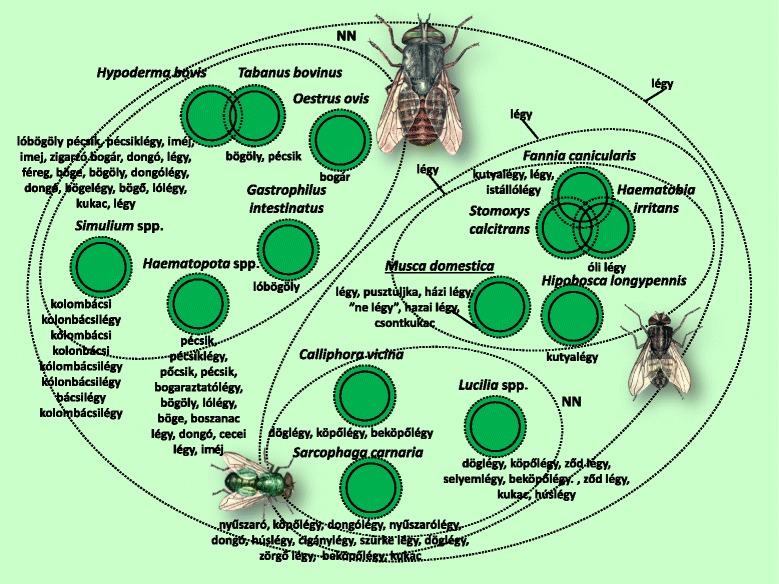
Folk taxa containing most of the true flies (Diptera)

**Fig. 6:**
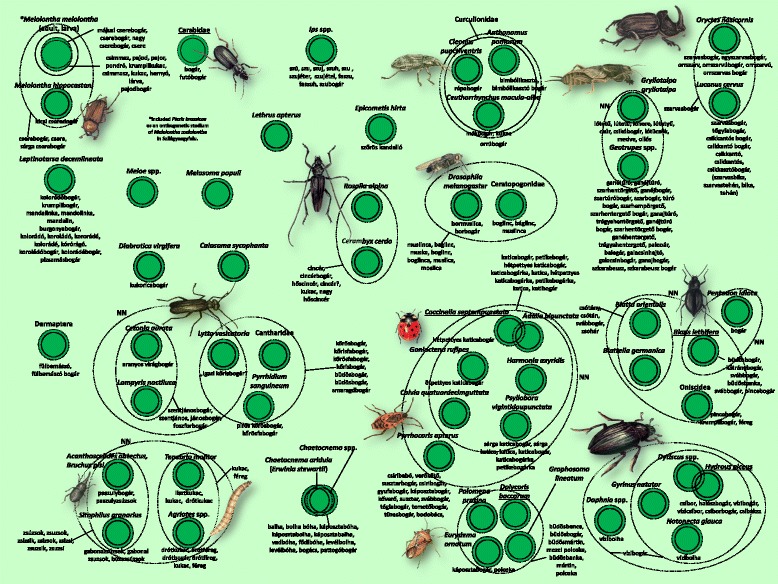
Folk taxa of the *bogár* (beetle) inclusive category, containing beetles (Coleoptera), as well as some true bugs (Heteroptera), crustaceans (Crustacea) and cockroaches (Blattodea), etc

**Fig. 7:**
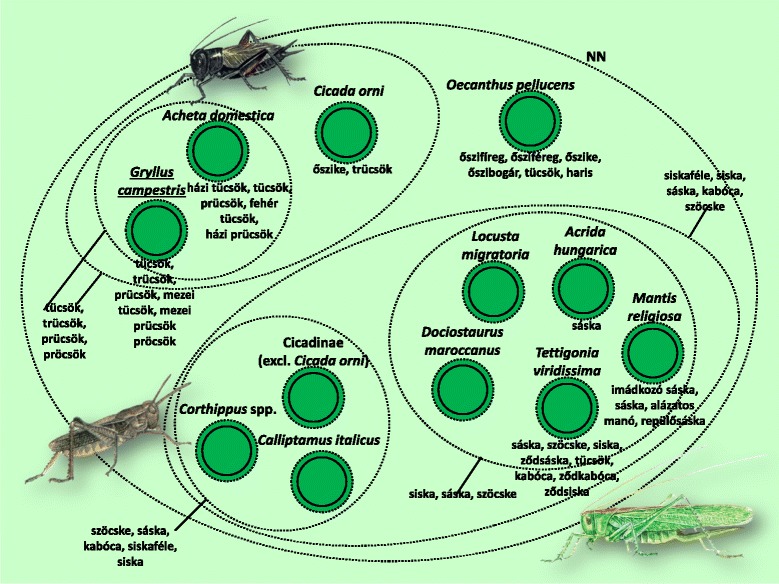
Folk taxa containing most grasshoppers, crickets and locusts (Orthoptera)

**Fig. 8:**
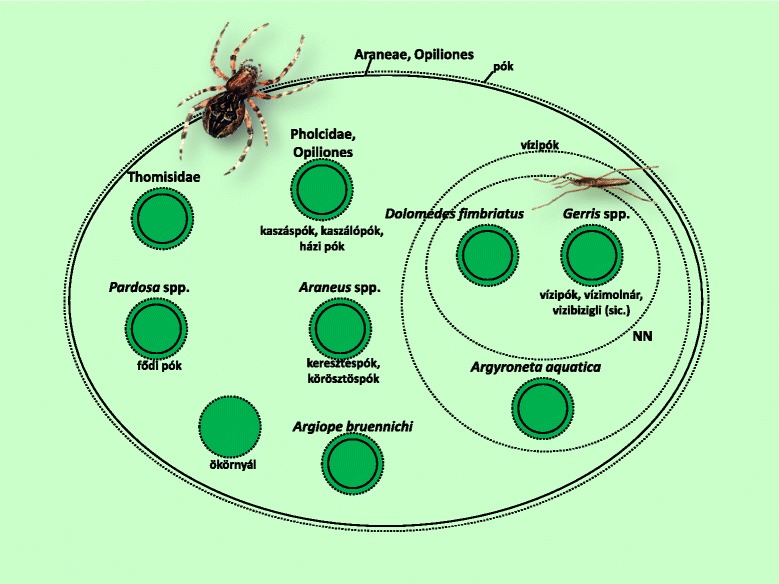
Folk taxa containing spiders (Araneae), and harvestmen (Opiliones)

**Fig. 9:**
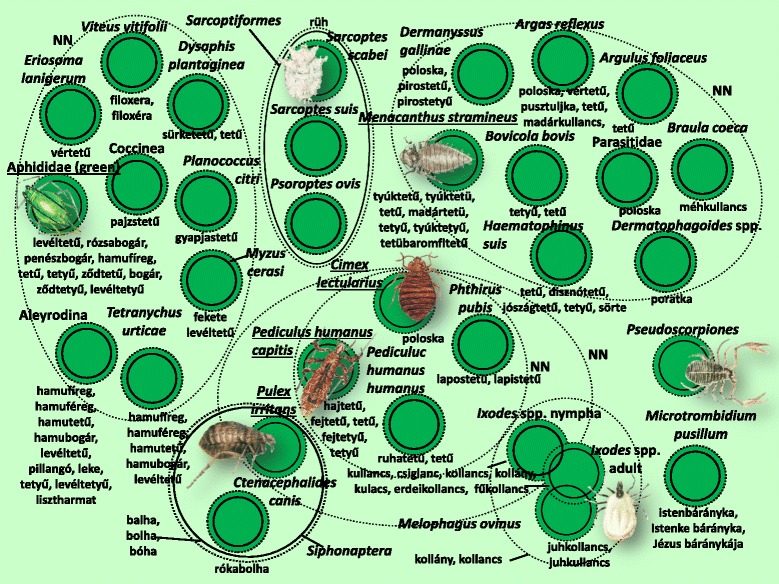
Folk taxa containing small parasites, herbivore pests and similar taxa

**Fig. 10:**
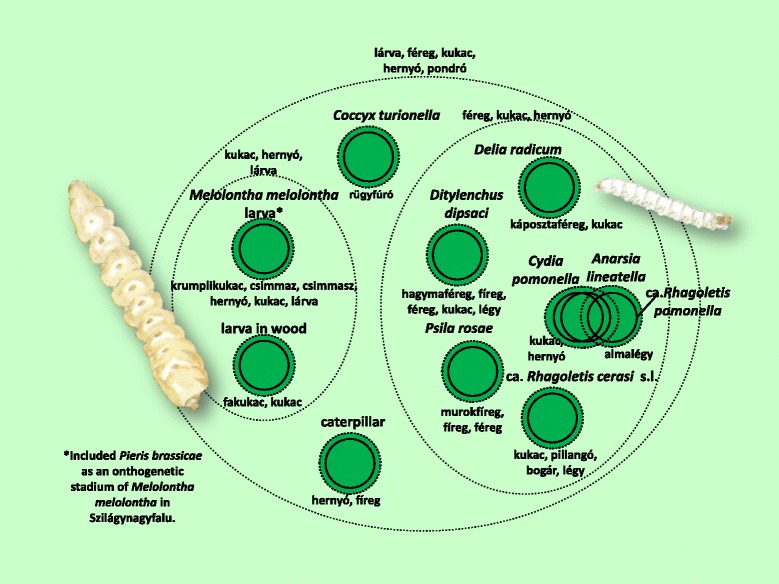
Folk taxa containing the larvae of some arthropods

**Fig. 11:**
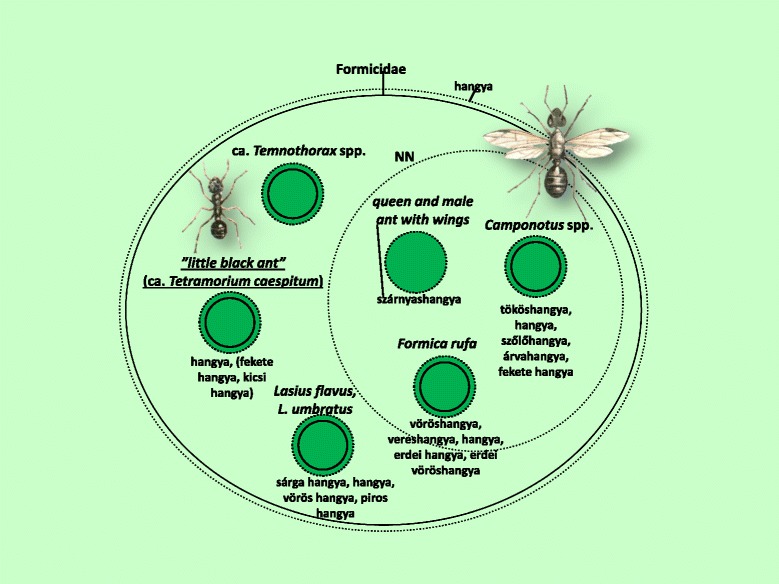
Folk taxa containing ant species (Formicidae)

**Fig. 12:**
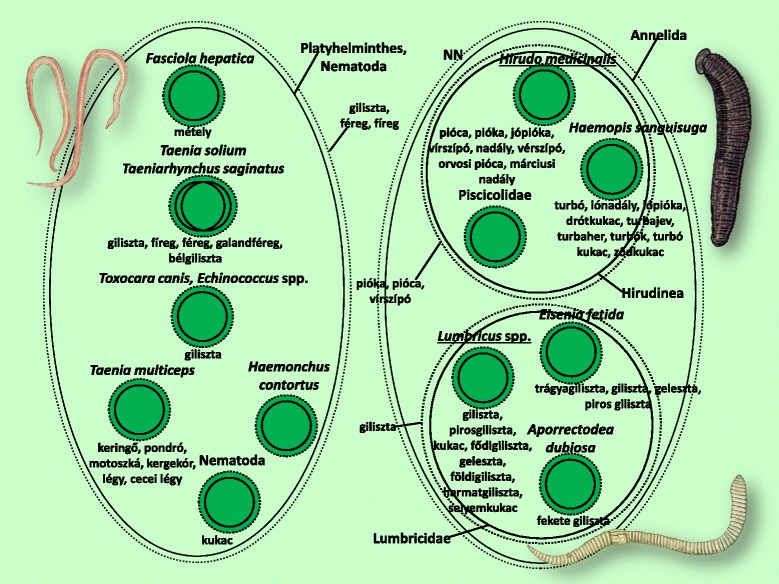
Folk taxa containing flatworms (Platyhelminthes), as well as roundworms (Nematoda), and ringed worms (Annelida)

**Fig. 13:**
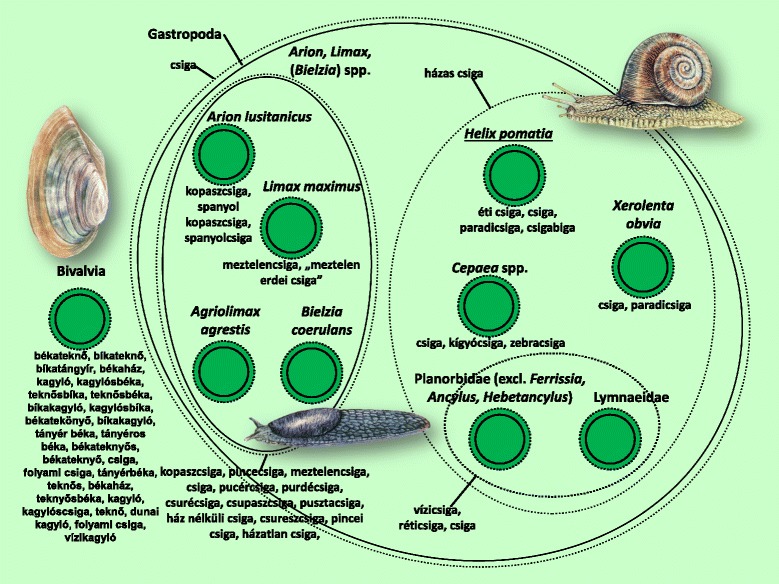
Folk taxa containing most of the molluscs (Mollusca)

**Fig. 14:**
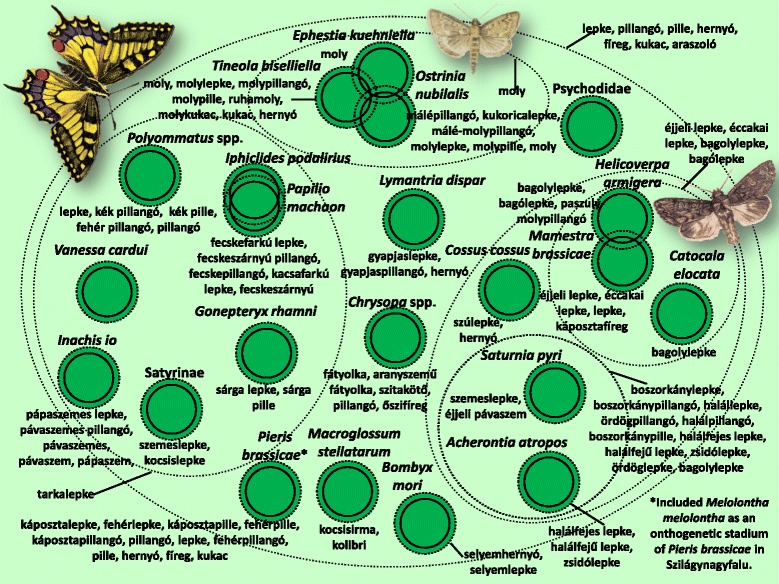
Folk taxa containing moths and butterflies (Lepidoptera) and some other taxa

The folk taxonomy and nomenclature for the 208 folk taxa are presented in Figs. 3, 4, 5, 6, 7, 8, 9, 10, 11, 12, 13 and 14. Further data (English equivalents, salient features, main habitats and proportion of people who knew the taxon) are contained in Appendix. 16 prototypic species have been recognised, sharing the following features: their names consisted mostly of one simple noun, and within each inclusive taxon they represented the most typical behaviour, were usually the most common species, or could serve as a basis for comparison due to some other feature.

The group containing all the hymenopteran taxa except for ants and gall wasps (Fig. 4) did not have its own separate name. Informants tended to divide this group into three parts: *méhek* (bees), *darazsak* (wasps), and *dongók* (bumblebees), the latter of which had a certain overlap with the *méhek* (bees) taxon. Prototypic species could only be identified for the first two, more stable groups.

The group called *légy* (fly) included a significant proportion of true fly (Diptera) species, and not a single group belonging to a different scientific taxon (Fig. 5). The dipteran folk taxa were distinguished primarily according to ecological salience, and secondarily according to morphological salience, into widely known taxa. The dipteran taxa *Fannia canicularis*, *Stomoxys calcitrans* and *Haematobia irritans* were not known to many informants, and could only be partly differentiated, never entirely. This state of uncertainty is reflected in the diagram with overlapping circles. We recorded knowledge of a total of 24 dipteran species, although informants did not include them all and always to the inclusive Diptera category.

The sole group to contain a large number of taxa was the one referred to as *bogár* (beetle or bug, *cf.* [45]), which totalled 48 folk taxa (Fig. 6). As with many of the inclusive folk taxa, there were no sharp divisions here either. With species that do not strictly belong in the group of beetles there were further instances of the name *bogár* (beetle) being used, but the species that feature in Fig. 6 are the ones that could be classified with greater certainty in the folk taxon of *bogár* (beetle). The key attributes for classification were the hardness of the integumentary system and the shape of the species. The most common taxa in this group were those with a hard chitinous covering and those belonging to the scientific order Coleoptera.

The folk prototypic species for the entire group of beetle (*bogár*) were primarily the black-coloured members of the family of ground beetles (Carabidae). The prototypic species for inclusive taxa with fewer members were the seven-spot ladybird (*Coccinella septempunctata*), the great silver water beetle (*Hydrous piceus*), and so on. There were examples of taxa at two separate levels being given the same name, even though the informants could clearly distinguish between the levels (see *vízibogár* [water beetle]).

The flea beetles (*Chaetocnema* spp.) constituted a special case. Here, the complex phenomenon was identified using a single taxon, the combined presence of a *Chaetocnema* species and an *Erwinia* bacterium species, which causes leaf dieback that forms a distinctive pattern.

Among ladybirds (Coccinellidae), informants could distinguish 5 or 6 species. The harlequin ladybird (*Harmonia axyridis*), a recently arrived invasive species, was almost universally known. In the year it appeared, this species was immediately noticed everywhere, and viewed as alien and harmful. The firebug (*Pyrrhocoris apterus*) is well known in every settlement, and has a wide variety of names (13 different names in the three areas).

Within the inclusive taxon of *bogár* (beetle), there were also instances of species with markedly different appearances (even to an untrained eye) being classified together. For example, the European mole cricket (*Gryllotalpa gryllotalpa*) was associated with the dor beetles (*Geotrupes* spp.), with the reason given that these species are found close to animal faeces.

The cockchafer (*Melolontha melolontha*) and its larva appeared in two (sometimes three) separate places within the folk taxonomy. In addition to the separation of the larva and the imago, the caterpillar of the large white butterfly (*Pieris brassicae*) (and, to a lesser extent, other species of butterfly) as well as its imago were regarded as stages in the ontogenetic development of the cockchafer. This was particularly true of the Sălaj area, although other scattered data [84] indicate that this view is common among much of the ethnic Hungarian population of Transylvania.

The folk taxon containing mostly orthopteran species only differed from the scientific classification in the absence of the European mole cricket (*Gryllotalpa gryllotalpa*). It did, however, contain the majority of cicadas (Fig. 7). The prototypic species in this taxon was the field cricket (*Gryllus campestris*). The distinction between this and the Italian tree cricket (*Oecanthus pellucens*), and therefore the entire classification as well, differed significantly among the different areas (in Sălaj all informants knew the distinction, but only one made the distinction in Gemer).

The harvestmen (Opiliones) and cellar spiders (Pholcidae) are different groups at ordinal level, but the informants treated them as a single folk specific (Fig. 8). The waterstriders (*Gerris* spp.), although belonging to the Heteroptera, were also included among folk spider species.

There was justification for classifying smaller parasites, plant pests and other similar species together (Fig. 9), although it was not possible to confine this group within an inclusive taxon that ruled out all uncertainty. The group was heterogeneous in terms of both the scientific taxonomy and the various folk saliences. The number of known species is high, and they were very accurately identified. There was a high number of taxa that had their own prototypic species [species of green aphid, chicken body louse (*Menacanthus stramineus*), bed bug (*Cimex lectularius*), head louse (*Pediculus humanus capitis*), human flea (*Pulex irritans*)].

Figure 10 shows the majority of the larvae of insect species. This was the most uncertain of the inclusive taxa, and was not regarded as an independent group by many of the informants.

Within the category of ants, there was one clearly defined folk taxon, namely the winged castes of the most diverse species of ant (Fig. 11).

Figure 12 shows the ringed worms (Annelida), flatworms (Platyhelminthes) and roundworms (Nematoda). The folk taxonomy of the ringed worms completely mirrored the scientific taxonomy, even at the level of two supraspecific taxa. In the case of the flatworms and roundworms, less information is available.

Within the molluscs, the group of snails and slugs was very clearly defined (Fig. 13). The bivalves sometimes shared associations with other molluscs (in their names, for example), but they were more frequently linked with frogs. Informants whose folk knowledge had suffered from the least amount of erosion almost exclusively regarded bivalves as the *eggs* of certain frog species (mostly *Pelophylax* and *Rana*).

Apart from the overlap with the cockchafer *(Melolontha melolontha)* and the special classification of lepidopteran caterpillars, the folk taxon of lepidopterans was also quite intact, and largely in agreement with scientific taxonomy (Fig. 14). Two additional folk taxa were included here which are classified differently according to entomologists: the moth flies (Psychodidae) and the lacewings (*Chrysopa* spp.).

**Fig. 15:**
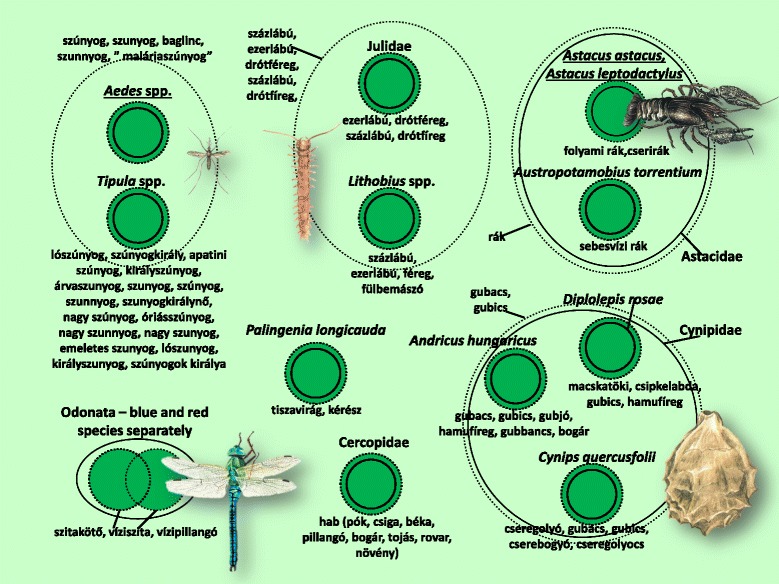
Smaller folk taxa containing other arthropods

Only a few invertebrate taxa were left out of all inclusive categories. Most of these remained alone during the pile sorting exercises. They could, on very rare occasions, be sorted into one group or other, although inconsistently, and without true conviction. Such taxa included e.g. the Tisa mayfly (*Palingenia longicauda*) and the froghoppers (Cercopidae) (Fig. 15).

Figures 3, 4, 5, 6, 7, 8, 9, 10, 11, 12, 13 and 14 show that 90 % of the taxa were embedded in the taxonomy, with generics and specifics dominating. The greatest degree of knowledge was connected to the more inclusive categories (and not to generics or specifics) primarily in the case of spiders, and to a lesser extent the snails, ants and lepidopterans. Berlin et al. [82] argued that biological species differ considerably in their overall distinctiveness from one another, and this differential distinctiveness leads to the formation of folk generic categories of differing degrees of perceptual importance. A significant part of the taxonomic literature, however, is about vertebrates, and the basic principles established in the literature often do not work with invertebrate groups. Among invertebrates, there is greater importance attached, for example, to prototypic species. These play an important role in taxonomic identification [81]. Nevertheless, the prototypic species were often given only brief descriptions by our informants. The reason for this may lie in the fact that these prototypic species were used as the basis for comparison. In such cases, the less typical species were the ones requiring more detailed descriptions, because they are being compared with and differentiated from the prototypic species.

Within a folk specific, we generally found species that were related from a scientific point of view. It was rare to find taxa that were far removed from each other according to scientific classification. As an example of the greatest distance, harvestmen (Opiliones) and cellar spiders (Pholcidae) (2 scientific orders) were identified as a single folk specific. The reason for this is probably because their physical structures are very similar (especially long legs). In line with previous findings [71], folk taxonomic relations were, to a significant extent, based on the morphological appearance of the taxa. The parallel with the scientific taxonomy was therefore surprisingly precise (especially in the case of ringed worms).

One interesting belief resulted in a quite remarkable taxonomy. The connection between bivalves and frogs is generally known in Sălaj, but was only reported by the most knowledgeable informants in Baranja. In the past, this knowledge may have been more widespread here as well. The connection between the two species is even reflected in the name of the bivalves (*békateknő* – “frog tub”). We could not find out how or where this belief originated. It is hard to perceive any axis on the bodies of the bivalves, so it could be that they were not regarded as an animal species in their own right for this reason. When touching the body of the bivalves, the experience is similar to touching the slimy skin of a frog, and furthermore, they live in the same habitat. Thanks to the media, and perhaps from speaking to relatives who have been to the seaside, many informants have now heard of seashells. The majority of these were called *kagyló* (shell), and they were sharply distinguished from freshwater species. More knowledgeable informants said that the seashells were, like their freshwater equivalents, the eggs of frogs. However, slight majority recognised that they are separate species. Several informants could identify tadpoles (one of the common folk names is *kutyahal* – “dogfish”), although surprisingly they were unaware of their relationship with fully grown frogs. Tadpoles therefore exerted no influence on the supposed link between bivalves and frogs. In Ghimeş (Gyimes, Romania), the tadpoles of the yellow-bellied toad (*Bombina variegata*) are used in veterinary medicine. The connection between the spawn (*tojás* - egg), the tadpole (*békapinty, frog carp?*) and the mature adult is recognised for all common species of frog occurring there [85].

Sometimes species were classified not (only) according to morphological salient features, but (also) ecological and cultural features (e.g. *Geotrupes* spp., *Gryllotalpa gryllotalpa*). In other words, species which are clearly different from each other, even to an untrained eye, could sometimes be placed into the same taxonomical group. In such cases, morphology, the default first priority when making classifications [71], was replaced by ecological differentiation.

A few species were included in the taxonomy which were not universally viewed as animals, with some informants describing them as diseases rather than species of fauna. These included the liver fluke and species of mite. This uncertainty may derive from the small size of the creatures, or from the fact that they are hard to observe. The small size of the animals involved may also be the main reason behind the various explanations given for the origin of “cuckoo spit” (meadow froghopper foam nests), *Erwinia* infestations of maize, and gossamer. These three phenomena were regarded as structures created by the most diverse range of species, and in the case of gossamer, several informants described it as a weather phenomenon.

Among the inclusive taxonomic categories, the one known as *bogár* [beetle] is closest to the “wug” taxon introduced by Brown [86]. “Wug” in the three regions studied included most invertebrate species, such as beetles, butterflies, bees and flies; it did not include molluscs, and only rarely did it also include flatworms, roundworms or ringed worms, so as a category it more or less covered the arthropods. The name *férgek* [worms], also often used as an inclusive category, was less readily applicable to the folk taxonomy. Sometimes the name was used for worm-like creatures, at other times it was applied to other invertebrate pests. In extreme instances, it even encompassed the house mouse, the wolf, the bear (*cf*. [63]), or indeed any animal regarded as harmful in any way.

### Human uses of invertebrate taxa

A total of 24 invertebrate species were documented as being of direct use to humans (Table 1). The use of invertebrates in our study areas was much less significant, than the role of plants in nutrition and medicine (e.g. [76, 87, 88]), or the role of insects in nutrition and medicine in other parts of the world [89, 90]. Four species were used for medicinal purposes, 5 species were consumed, 11 were used as bait for fishing, and 2 species were used as playthings. Compared with the tropics (27 medicinal species [91]; more than 200 edible species [92]) this is much lower both in diversity and in terms of the body mass of the invertebrates used.

**Table 1:** List of invertebrate taxa for medicinal use, food, angling, toy and other purposes. The last column indicates which species are traditionally protected by locals

	See taxonomy in Fig. no.	Latin and proper name of folk taxa (serial number in the appendix)	Saliences	Medicinal	Consumption	Bait	Toy	Other usage	Protection
1.	15	*Andricus hungaricus* Hungarian gall wasp (132)	*Used for tanning, but collected here mainly for sale.*				x	x	
2.	13	*Arion, Limax* spp. e.g. *Limax maximus* slug species (14)	*They are very good for baits (i.e.: for angling).*			x			
3.	4	*Xylocopa violacea, Xylocopa valga* black coloured carpenter bees (112)	*Eats nectar, it doesn’t do you any harm. / Drills the wood like a machine. / We frequently caught it, took apart and ate the honey from it.*		x				
4.	9	*Microtrombidium pusillum* dwarf velvet mite (46)	Sometime it is protected like a taboo. *God’s Lamb. It has a cross on its back.*						x
5.	6	*Lytta vesicatoria* Spanish fly (86)	*If the rabid dog bit someone, you had to feed nine piece of it to the man. / If you pour (the tincture prepared from the beetle) onto the head of the man, he did not shiver any more.*	x				x	
6.	6	*Pyrrhidium sanguineum* Welsh oak longhorn beetle (92)	*We would use them for fishing long time ago.*			x			
7.	13	*Helix* spp. mainly *Helix pomatia* edible snails (20)	*The poor cooked it. / They were collected in springtime. / They were washed at least ten times. It was scalded and the foot cut off. It was soaked in lukewarm water, in cold water, lukewarm again, a lot of work. / Snails are best before the weeds grow too high.*		x				
8.	15	*Astacus astacus, Astacus leptodactylus* European crayfish, Danubian crayfish (48)	*The old of long time ago caught it, it became red when cooked. / My father caught many on the Rét* (a marsh)*, we cooked them in a big pot. In salty water. / The tail and the nippers are good to eat.*		x				
9.	8	Araneae e.g. *Tegenaria domestica* spiders (34)	*If you cut your feet, you would pick spider net in the stable and covered the cut to heal.*	x				x	x
10.	4	*Apis mellifera* European honey bee (113)	*When the bees are gone we will be gone as well because there will be nothing to eat. / Honey is good for a lot of things.*	x	x			x	
11.	13	Bivalvia e.g. *Anodonta cygnea* clams (24)	*There were many, fed to the pigs. / You would make buttons of it. It is good for bait to catch carp and predatory fish.*			x		x	
12.	12	*Hirudo medicinalis* European medicinal leech (8)	*My aunt had them in a jar, when she had a headache or neck ache you would put them on. / Only March leech would be good. / If your tooth aches, put to your gums, it would suck the bad blood from it.*	x		x			
13.	12	*Haemopis sanguisuga* horse-leech (10)	*It’s like the leech but only more gentle. / We would pick them to catch catfish.*			x			
14.	12	*Lumbricus* spp. e.g. *Lumbricus terrestris* earthworms (11)	*My husband would know them because he was a fisherman and would collect them.*			x			
15.	12	*Eisenia fetida* redworm (12)	*Not all earthworms would do for angling. This is the best one.*			x			
16.	12	*Aporrectodea dubiosa* earthworm species (13)	*This is harder and it* (the fish) *can not pull it down* (from the hook).			x			
17.	6	*Daphnia* spp. e.g. *Daphnia magna* water fleas (50)	*There was a doctor here when we were kids who had an aquarium and he gave them to the fish. We would go to collect them with a little dipping net.*					x	
18.	6	*Gryllotalpa gryllotalpa* European mole cricket (63)	*This is a good bait* (to angle)*. / They were gathered to put on bottom hooks, there were some 200 bottom hooks attached on a single string.*			x			
19.	6	*Cerambyx cerdo* great capricorn beetle (90)	*I would pick them out for bait* (from firewood). *In winter, when I can’t get earthworms.*			x			
20.	-	different beetles and other bigger insects	cruel playing with living individuals				x		
21.	6, 10	*Melolontha melolontha* cockchafer (88)	*You can angle with it nicely. When it has time* (swarming)*, fishes like it.*			x			
22.	6	*Coccinella septempunctata* seven-spot ladybird (97)	*We are scared that they* (*Harmonia axyridis*) *will kill off all of our nice little ladybugs. Oh, those littles. Which is a pity, because they are good.*						x
23.	4	*Osmia adunca* mason bee species (115)	*We picked out the reed* (from the roof)*, when we saw that there was reed honey in it. My grandmother was very angry and scored at us because we destroyed the reed roof beehive and we then ate the reed honey.*		x				
24.	15	*Cynips quercusfolii* gall wasp species (130)	*We were kids and made pipe of it. It was a toy. / Oak galls would be used for tanning leather in the past.*				x	x	
25.	14	Lepidoptera e.g. *Melitaea athalia* butterflies (135)	*This is indeed not a pest. We were glad to see it before.* They are aware of the harm many species do, yet adult individuals are not destroyed.						x

*Andricus hungaricus* and *Cynips quercusfolii* were known to be used for tanning leather, although rarely. Even less commonly, *Lytta vesicatoria* was mentioned as an aphrodisiac, and *Daphnia* spp. were used as food for aquarium fish.

Completely new was the discovery that the honey stomachs of black-coloured carpenter bees (*Xylocopa violacea*, *X. valga*) were consumed even when there was no shortage of alternative foods. This practice was previously unknown in Europe.

In the last hundred years, the consumption of invertebrates in Europe has traditionally been restricted to just a few species [93], and in the areas of our investigation, they were only consumed occasionally. The consumption of *nádiméz* (honey in the tube of the reed) from thatched roofs was quite widespread among children, but the decline of this practice may be due to the gradual replacement of thatching as a roofing material. Molluscs are consumed relatively commonly across the globe [14, 26], and this was also true for the three study areas in the past [94]. Surprisingly, the idea of consuming edible snails was mostly rejected as disgusting by the informants, and it was only among the most elderly informants in Baranja that there was any tradition of eating edible snails.

The use of Spanish fly (*Lytta vesicatoria*) was well known, although very few informants had actually seen it used in practice. Its consumption was sometimes linked to superstitious elements such as consuming a “magic number” (9) of beetles placed in palinka (distilled fruit spirit), and mixing them with “randomly” found dog faeces. Blister beetles are used the world over to treat incurable or barely curable illnesses [95], and in our study areas, they were previously used as an antidote to rabies.

We found that leeches were used in four ways: 1) placed on the neck to reduce blood pressure, 2) for treating symptoms of periodontitis, 3) as a painkiller, by increasing the flow of blood as well as from the analgesic entering the bloodstream, 4) as a fishing bait. One species (European medicinal leech - *Hirudo medicinalis*) has a medicinal effect, while the other (horse-leech - *Haemopis sanguisuga*) does not. Detailed morphological knowledge was of great importance here. In the Carpathian Basin, it is common for medicinal and non-medicinal plant species also to be given the prefix of *orvosi* (medicinal) or *ló-/kutya-* (horse/dog), respectively [75, 76].

The use of invertebrate taxa for veterinary medicine was not documented in any of the areas under investigation, although such practices are known in the region. In north-east Romania, for example, spiders are used to treat flatulence in cattle by rubbing the spider into the animal’s side [Ulicsni ined.].

Contrary to our expectations, we did not document any current uses for blister beetles or slugs. The use of slugs as a lubricant of cartwheels [51] was not mentioned in our study areas. Based on other data collections, however, this practice was known in the Carpathian Basin [Molnár ined.].

### Proverbs and sayings

Invertebrates are featured in a number of proverbs and sayings (Table 2). During data collection, a total of 30 taxa were associated with a proverb or some other folk wisdom (such as weather forecasting, harvest predicting, similarly to the way in which birds, for instance, are associated in many human cultures [96]). Some of these were based on observations of animal behaviour or experience of their population cycles, and so do have some genuine basis in fact (e.g. the swarming patterns of *Lytta vesicatoria*). Other folk beliefs, however, were probably closer to old wives’ tales (e.g. drawing a cross on the back of a field cricket will prevent it from jumping; the presence of *Andricus hungaricus* prevents hens from brooding). Forecasts of weather phenomena based on the behaviour of various invertebrates (e.g. winged ants mean that rain is coming) occurred frequently.

**Table 2:** Proverbs and common sayings referring to Invertebrata

Latin name, proper name (serial number in the Appendix)	Proverbs, their meanings and explanations
*Coccinella septempunctata* seven-spot ladybird (21)	*We said to it: ladybug, where do you take me to get married? Then we married in the direction where it flew.*
	Vernacular prophecy.
Planorbidae (excl. *Ferrissia, Ancylus, Hebetancylus*) e.g. *Planorbis planorbis* ramshorn snails (23)	*If the snail climbs up from water onto something, it means the flood is coming.*
	Vernacular prophecy.
Gastropoda (excl. slugs) e.g. *Zebrina detrita* snails (25)	*Snail. This is the strongest animal carrying its house on its shoulder.*
	Joking comparison frequently quoted to kids.
*Ixodes* spp. e.g. *Ixodes ricinus* ticks (28)	*You’re like a tick.*
	Said mostly to kids with an affectionate joking gesture because of their attachment.
gossamer air-threads (44)	*There will be no rain because it stretches.*
	Vernacular weather forecast.
*Microtrombidium pusillum* dwarf velvet mite (46)	*Shine, sun, shine, Jesus’ lamb is freezing to death under the gardens. And then the Sun shone.*
	A superstition wishing to change the weather.
*Oecanthus pellucens* Italian tree cricket (55)	*Autumn is here because the cricket chirps, saying ’gather, gather’.*
	Wisdom based on observations impersonating the species.
*Gryllus campestris* field cricket (61)	*You could not put down your clothing in the grass because old people said: the cricket would gnaw a hole in it.*
	Might be a belief.
*Gryllus campestris* field cricket (61)	*If you draw a cross on the back of a cricket, it would not jump any more.*
	Fun for kids based on belief.
*Pyrrhocoris apterus* firebug (64)	*They stick together like the firebug.*
	The firebug (*Pyrrhocoris apterus*) can be seen in dense masses in springtime.
*Lytta vesicatoria* Spanish fly (86)	*The ash tree is stinky, it will rain.*
	Prophecy connected to Spanish fly invasion.
*Melolontha melolontha* cockchafer (88)	*If there are too many maybeetles, corn yields will be good.*
	Maybe vernacular experience or possibly only a belief.
*Cerambyx cerdo* great capricorn beetle (90)	*Your moustache stands up like that of a capricorn beetle.*
	An analogy on the long moustache bending upwards.
*Apis mellifera* and Araneae European honey bee (113), spiders (34)	*Bees collect honey, spiders poison from the same flower.*
	Meaning of the proverb: there is no universal truth.
winged ant castes (120) e.g. *Tetramorium caespitum*	*When the winged ant comes out, it will rain.*
	Vernacular weather forecast.
*Tetramorium caespitum* and similar species pavement ant (124)	*Be like the ant and work!*
	Ants were considered ‘diligent’ animals (busy as an ant).
*Vespa crabro* European hornet (127)	*Nine hornet bites kill a horse.*
	Based on real observation, augmented to mythical heights (9 is a mythical number in Shamanism).
*Vespula vulgaris* and similar species common wasp (128)	*Yellow wasp, small wasp, large wasp, they all scratch on a bunch of grapes.*
	Pun made of a vernacular observation.
*Andricus hungaricus* Hungarian gall wasp (132)	*My mother made us thrown them away. You must not keep it at the house because brood will not hatch the eggs.*
	Belief says it prevents brooding of the hen.
Lepidoptera e.g. *Melitaea athalia* butterflies (135)	*The superstition was that if you see a yellow butterfly in spring, you would fall ill. If you see a red one, you will remain healthy and fall in love, if a black one, someone would die.*
	Vernacular prophecy. The yellow butterfly may be *Gonopteryx rhamni*, red ones may be several other species.
Lepidoptera e.g. *Melitaea athalia* butterflies (135)	*Even the mottled butterfly came from a caterpillar.*
	You do not necessarily worth more just because of your better appearance or even something ugly may become beautiful one day.
*Saturnia pyri* giant peacock moth (151)	*The boszorkánylepke* (witch butterfly) *were nailed above the door for superstition.*
	It was used as a superstitious protection against the Devil.
*Drosophila* spp. e.g. *Drosophila melanogaster* fruit flies (159)	*Fruit flies cause the wine to ferment.*
	In their opinion the presence of fruit flies cause the wine to ferment.
*Drosophila* spp. e.g. *Drosophila melanogaster* fruit flies (159)	*The man from Vörösmart swallowed the frog; he thought it was a fruit fly.*
	Mocking a village.
*Tipula* spp. e.g. *Tipula maxima* crane flies (160)	*We stroke the mosquito king to death; there will be no mosquitos now.*
	The *Tipula* species which are much greater than biting mosquitos but are related to them are presented by the saying as a kind of king.
*Musca domestica* housefly (169)	*Noah wanted to chase them out from the Bark. He could not. Well, fly, then. He said. And the name stuck.*
	Folk etymology for the name of the fly. He blames Noah for the existence of flies. *Légy* in Hungarian also means: be (you should exist).
*Musca domestica* housefly (169)	*If flies bite, rain comes.*
	Vernacular weather forecast.
*Pediculus humanus capitis* head louse (191)	*It’s not a shame to get it, only to keep it.*
	Educating saying on responsibility.
*Haematopinus suis* hog louse (193)	*You can find a louse only in a good hog.*
	In their opinion louses occur on healthy pigs only.
Odonata e.g. *Sympetrum sanguineum* dragonflies (207–208)	*Where there are dragonflies, there are no snakes.*
	It was held that wherever a dragonfly hovers over the water there will be no snakes in it.

The positive attitude towards the presence of hog lice on swine is probably based on the observation that parasites abandon sick or dead livestock. Gub [61] also found examples of healing involving external animal parasites, a practice that can also be deduced from the same kinds of observation.

Games with the invertebrates, and the ill-treatment of animals were quite widespread in the past, although they were not confined to particular species. Nevertheless, larger and more easily caught species, such as *Melolontha melolontha*, *Lucanus cervus* and *Oryctes nasicornis*, were more likely to fall victim. Gub [61] describes several special games involving the cockchafer and the stag beetle.

In addition to Vallejo and González [55], Gub [61] also mentions the use of head lice in human medicine, especially in treating jaundice. We did not document any similar instances, although this practice may well be widespread, and with further research there is a high chance of finding more such cases.

One belief that made a scattered appearance in the areas under investigation stated that a dragonfly hovering about the water indicated that there was no snake in the water. The name recorded for the dragonfly by Gub [61], *kígyópásztor* (snake-shepherd) may also derive from this belief.

### Invertebrate species that enjoy folk conservation or state protection

Conscious ideas about conserving invertebrates only occurred with a few taxa (see the last column in Table 1). Seven-spot ladybirds, dwarf velvet mites and often spiders were said to enjoy protection, but informants would generally – but not universally – refrain from harming firebugs, field crickets and most butterflies.

With regard to ladybirds, the tradition of protecting them came from the culture (songs and sayings), but they were also recognised as useful animals. Many informants knew that they help reduce aphid populations. The taboo about destroying dwarf velvet mites was explained by a few informants as being due to the cross-shaped marking on their backs. Many stated that hurting spiders brought bad luck.

Butterflies were respected for their beauty. Here it should be noted that the state protection enjoyed by certain species of butterfly (e.g. *Iphiclides podalirius*, *Inachis io*) in Hungary is justified more by their beauty than their rarity.

Field and house crickets were generally left unharmed as a result of their pleasant chirruping and their cultural significance. Surprisingly, most people knew nothing about legal protection for invertebrates.

Also surprisingly, almost every invertebrate species was regarded as basically harmful. Where possible they were destroyed or at least regarded as being worth eradicating. Informants reported little information about the benefits of invertebrates, or did not regard the benefits as significant. Because they are very common, even species that were regarded as useful were not given any protection (for example, fruit flies are believed to aid fermentation). However, we could not find any information to suggest that any invertebrate species had disappeared or become rarer as a result of conscious destruction.

In the areas under investigation, traditional uses of and attitudes towards invertebrates have not revealed any kind of activity that would cause major damage from a nature conservation point of view. The fundamental factors behind this state of sustainability are small-scale farming, which imposes less strain on the environment, and the fact that resources are mostly used locally. Traditional methods of agriculture do without chemicals, so populations of many invertebrate species only began to decline as intensive farming spread (starting in the 1980s).

With the exception of edible snails and in a few cases certain galls the use of invertebrate taxa had remained local, and was therefore sustainable. In areas where the use has spread beyond the locality, for example in Mexico, with invertebrates living in species of *Agave* [90], or in areas of the Carpathian Basin where edible snails are harvested in big quantity [64], a significant reduction in the prevalence of such species has been experienced. The effects of such destruction have tended to be far more significant with regard to vertebrate taxa ([7], e.g. predatory mammals and birds).

### Folk wisdom related to nature as a whole

Sometimes knowledge pertaining to the taxa could have a more general relevance, and be regarded as folk wisdom concerning the functioning of nature as a whole. The damage caused by the gypsy moth (*Lymantria dispar*), for example, was regarded as a minor problem, because – according to many informants – major damage does not occur by itself “in nature”, only as a result of human intervention. A kind of tolerance was exhibited, especially in connection with species that people were fond of whatever reason, or regarded as relatively harmless, in phrases such as “they have to eat too”, or “they are also God’s creations”. The damage caused by such species is often accepted, and regarded as tolerable and natural. Certain instances of “wisdom” appeared not to originate from traditional folk knowledge. The view that “if the bees disappear, then we will disappear too, because there won’t be anything to eat” probably springs from the influence of the media.

Folk wisdom in our study areas was fragmentary, probably heavily eroded, and seemed no longer to constitute a unified, systematic world view, or social conventions that impact on everyday behaviour and thinking, as has been described e.g. in connection with the ontology of Native Indian communities in North America [97–99].

## Conclusions

Despite the fact that our material was gathered only recently, folk knowledge is still alive among Hungarian people in these regions, as are some of the folk uses. We argue, however, that before the dual impact of the market economy and public education became so powerful, Hungarian rural people might have possessed knowledge as deep as that of, for example, the natives of Amazonia. Ethnographic works from the late 19th and early 20th centuries provide the basis for this argument. The high number of known invertebrate folk taxa documented in our three study areas suggests that it would be worth conducting further investigations in other areas of Europe as well.

Local traditional ecological knowledge of invertebrates is highly relevant to helping us understand the mentality and worldview of local people. Understanding local worldviews can be a first step towards developing locally appropriate, culture-specific nature conservation strategies and local school curricula – desperately needed in our globalising world.

## Appendix

For each of the taxa, we collected and documented the local name (or names), their salient features, their uses, any damage they cause, any personal attitudes expressed towards the taxa (positive, negative or neutral), and related folklore issues. The habitats of the species were determined based on our own experiences, on the interviews, and on the scientific literature. Literal quotations are in italics, and comments by individual interviewees are separated by a slash.

**Table 3:** Data base of invertebrate folk knowledge among Hungarians

	Fig.	Scientific and proper names	The most typical local names and their literal English translation	Saliences	Key places of encounter and habitats	Proportion of informants who knew the taxon (%)
1.	12	*Fasciola hepatica* common liver fluke	*métely*	*It is in the liver of the livestock. It’s like a pumpkin seed cut in half. Fluky stock is skinny. / You must not graze it around lakes. Surely some snail spreads it.*	P	64
2.	12	*Toxocara canis Echinococcus* spp. dog roundworm	*giliszta*	-	P	9
3.	12	*Taenia solium Taeniarhynchus saginatus* pork tapeworm, beef tapeworm	*giliszta, galandféreg* (galandworm)	*It can be found in pigs, piglets, the guts, even in man, as big as half a metre long was also taken out.*	P	38
4.	12	*Taenia multiceps* tapeworm species	*keringő* (whirler)*, motoszká* (fumbler)	*A fly lays the egg into the nostrils of the sheep and it goes up to the brain. When it is developed there, the sheep would blow it out. If one does not blow it out, it will get the circling disease. / Before, we would operate them.*	P	4
5.	12	Nematoda e.g. *Pseudocapillaria tomentosa* fish roundworm species	*giliszta*	*These worms like the sterlet (Acipenser ruthenus) very much, they get into the stomach. It is thin like a needle.*	P	2
6.	10	*Ditylenchus dipsaci* stem nematode	*fíreg, kukac*	*Onions get worms as well. Small little worms. Yellowish.*	S	9
7.	12	*Haemonchus contortus* barber’s pole	*(piros) féreg* (red féreg)	*The cow has that manyplies, it was all full with red worm inside.*	P	2
8.	12	*Hirudo medicinalis* European medicinal leech***	*pióka, vérszípó* (blood sucker), *nadály*	*We would go into the water and it stuck on our legs. It was collected. / We would sprinkle ash on it and parted with the skin. It lives long. There are people who’s blood it does not like. / The leech is not a parasite; it was used for medicine centuries long.*	P, W	100
9.	12	Piscicolidae e.g. *Piscicola geometra* leech species on fishes	*pióca*	*They kill the fish; suck their blood, stuck on them.*	P, W	5
10.	12	*Haemopis sanguisuga* horse-leech	*lópióka* (horse pióka)*, turbók, drótkukac* (wire worm)	*It was dug out from wet earth. / We call it the wire worm. They are this big and hard, dark green. /*	W	80
11.	12	*Lumbricus* spp. e.g. *Lumbricus terrestris* earthworms	*giliszta, földigiliszta* (earth giliszta)	*Selyemkukac* (silkworm) *are in the garden, around the house, under the bricks, after rain, they breathe in the fresh air. It’s a soft bodied worm.*	S	100
12.	12	*Eisenia fetida* redworm	*giliszta, trágyagiliszta* (dung giliszta)	*It is beside the dung. / Reddish. Not so big.*	S	10
13.	12	*Aporrectodea dubiosa* earthworm species	*fekete giliszta* (black giliszta)	*It is on the waterside. Black.*	W	2
14.	13	*Arion, Limax* spp. e.g. *Limax maximus* slug species	*kopaszcsiga* (bald snail), *meztelencsiga* (naked snail), *csupaszcsiga* (nude snail)	*It ate members of the cabbage family. / You could hardly find a plant which would not be damaged by them. / It is usually found such dark cellars. Wherever it goes, leaves this discharge behind. / After the rain. / I draw them from the well.*	A, S	96
15.	13	*Agriolimax agrestis* and similar species smaller field slugs	*meztelencsiga* (naked snail), *kopaszcsiga* (bald snail)	*The white ones come in every four or five years but would then teem frightfully.*	A	4
16.	13	*Arion lusitanicus* Portuguese slug	*kopaszcsiga* (bald snail), *spanyol kopaszcsiga* (Spanish bald snail)	*They are visitors here. You would not find them long ago. / It will spread here as well. / It came from Spain with vegetables and are very prolific.*	A	16
17.	13	*Limax maximus* great grey slug	*meztelencsiga* (naked snail), *meztelen erdei csiga* (naked forest snail)	*They would gnaw away mushrooms instantly. / They would eat it, whether edible or poisonous.*	F	5
18.	13	*Bielzia coerulans** Carpathian blue slug	*meztelencsigá* (naked snail)	*You can find blue or grey ones.*	F	5
19.	13	*Cepaea* spp. e.g. *Cepaea vindobonensis* land snail species	*csiga, kígyócsiga* (snake snail)	*This was called the snake snail. Where the name does come from I have no idea. / They collect the dew drops.*	A, S	14
20.	13	*Helix* spp. (*) mainly *Helix pomatia* edible snails	*csiga, éti csiga* (edible snail)	*This is the strongest animal because it carries its house on the back. / I would not do any harm to them, even though they can make trouble. / I always tread on them. They like to eat my flowers. I would throw them back to the hens. / They are not so harmful.*	G, S	100
21.	13	*Xerolenta obvia* land snail species	*csiga, paradicsiga*	*These are white little snails on the plants. They would also stick to the grass leaves.*	G, S	55
22.	13	Lymnaeidae e.g. *Lymnaea stagnalis* freshwater snail species	*vízicsiga* (water snail)	*During floods (high water) they climb on boats or a thick branch. Floods are coming when the snail climbs out of the water.*	W	6
23.	13	Planorbidae (excl. *Ferrissia, Ancylus, Hebetancylus*) e.g. *Planorbis planorbis* ramshorn snails	*csiga, vízicsiga* (water snail)	*When the water was rising, this came up to the surface.*	W	12
24.	13	Bivalvia e.g. *Anodonta cygnea* clams	*békateknő* (frog tub), *kagyló*	*Frog tub. We would pick them when I was a kid. It comes off from the frog. Like the egg from the inside.*	W	83
25.	13	Gastropoda e.g. *Zebrina detrita* snails	*csiga*	*They would gnaw during the night and they drag that mucus behind. / Little snail come out, your house is burning. You’ll get milk and butter, it will be left for tomorrow*. (a child song)	F, G	100
26.	15	Julidae e.g. *Megaphyllum unilineatum* millipede species	*ezerlábú* (thousand legged)*, drótféreg* (wire worm)	*I have seen this little black insect on the garbage heap. / Who’s got the patience to pick up so many of them? They would have swept them, obviously.*	S, G	16
27.	15	*Lithobius* spp. e.g. *Lithobius forficatus* common centipedes	*százlábú* (hundred legged)	*It’s so reddish. / You can get many of them when you lift the flower pots.*	S, G	44
28.	9	*Ixodes* spp. e.g. *Ixodes ricinus* ticks	*kullancs, csiglanc*	*I think they are not infected here. They are rather on the blades of grass. It is dangerous because it spreads encephalitis. / You would pick at it or you put fat or oil on it and than it would climb out or fall out. / The one living on animals would not get into humans. / You would say to little kids ‘you’re a tick’. / It was not dangerous before. I think this has become infested due to this many poisons and the atom.*	P, F	94
29.	9	*Dermatophagoides* spp. e.g. Dermatophagoides *pteronyssinus* house dust mites	*poratka* (dust atka)	*-*	H	2
30.	9	*Sarcoptes scabiei* itch mite	*rüh* [it is not seen as an animal]	*It would creep into your skin and little pimples would appear. It would also get wedged in among the fingers. / Something was mixed in pig fat and used as ointment.*	P	35
31.	9	*Sarcoptes suis* pig mange mite	*rüh*	*When piglets got the itch, they would be smeared with fat, nowadays with cooking oil.*	P	9
32.	9	*Psoroptes ovis* sheep scrab	*rüh*	*The Temoxa, we would dip them in summer and then their wool would not fall out.*	P	7
33.	9	Pseudoscorpiones e.g. *Chelifer cancroides* false scorpions	no name	*This is a little beetle, I can see them some times. They are like the ones in the TV (*scorpions*), only they are little. It fell from a tree. It has two feelers.*	H	5
34.	8	Araneae e.g. *Tegenaria domestica*spiders	*pók*	A wide spread belief says spiders must not be killed because it brings misfortune.	H	100
35.	8	*Dolomedes fimbriatus** raft spiders	*vízipók* (water pók)	*The same shape as a spider.*	W	9
36.	8	*Argyroneta aquatica** diving bell spider	*vízipók* (water pók)	*It’s got a big bladder* (in fact, a bubble) *with which it goes down.*	W	4
37.	8	*Araneus* spp. e.g. *Araneus diadematus* spider species	*keresztespók* (crossed pók)	*You put it into a white bag and let it out in the morning. You would open the bag and it has written your fortune numbers there. / We were afraid of them because they stung. / It is Greek Catholic because it’s got a double cross.*	G	42
38.	8	*Argiope bruennichi* wasp spider	*pók*	*This is like a guest spider in these parts. / But it did not eat the common wasp.*	G	5
39.	8	Pholcidae e.g. *Holocnemus pluchei* Opiliones e.g. *Phalangium opilio* cellar spiders and harvestmen	*kaszáspók* (scything pók)*, házi pók* (house pók)	*You pick its leg out, it would still work for a while, sawing the air. / You get plenty of them in the villages.*	H	61
40.	8	*Pardosa* spp. e.g. *Pardosa alacris* wolf spiders	*fődipók* (ground pók)	*Ground spider* (that is: not a net weaving species). *It has eggs on the back.*	O	5
41.	8	Thomisidae e.g. *Thomisus onostus* crab spiders	*pók*	*You can get yellow ones as well. Sits on flowers.*	G	4
42.	9	*Dermanyssus gallinae* poultry mite	*poloska, pirostetyű* (red louse)	*It’s there right away in tiny chicks. / You must roast onions and smear it under their little wings, at the tail and the neck. Or, they are stamped out with smoke.*	P	9
43.	9	*Argas reflexus* pigeon tick	*madárkullancs* (bird tick), *vértetű* (blood louse)	*It is very quick. If it spreads in poultry, it would suck their blood, there is plenty of them.*	P	11
44.	8	gossamer	*ökörnyál* (ox saliva)	*There will be no rain because the gossamer is stretching* (i.e. carried by the wind)*. / It usually flies during Indian summer.*	G	16
45.	9	Parasitidae e.g. *Parasitus coleoptratorum* a family of predatory *mites*	*poloska*	*A tiny red bug.*	P	5
46.	9	*Microtrombidium pusillum* dwarf velvet mite	*Istenbárányka* (God’s lambkin), *Jézusbárányka* (Jesus’ lambkin)	*It’s so velvet-like, beautiful, no dresses like it are ever made. /* They sang: *Shine, Sun, shine, Jesus’ lambkin freezes to death under the gardens. And then the Sun shone. / You could see it in springtime.*	S	17
47.	9	*Tetranychus urticae* red spider mite	*hamuféreg* (ashféreg)	-	A	4
48.	15	*Astacus astacus*, Astacus leptodactylus** European crayfish, Danubian crayfish	*rák, folyami rák* (river rák), *cseri rák* (tanned rák)	*It’s on the water bottom, on pebbles. / Once upon a time our canals were so clear, full of crabs.*	W	90
49.	15	*Austropotamobius torrentium* Stone crayfish	*sebesvízi rák* (rapid waters crab), *rák*	*This is upstream, in mountain creeks. / You can’t eat it because it’s so tiny.*	W	2
50.	6	*Daphnia* spp. e.g. *Daphnia magna* water fleas	*vízibolha* (water flea)	*Very little, bouncing in water.*	W	2
51.	9	*Argulus foliaceus* common fish louse	*tetű* (louse)	*You can find it in marshy lands. / Fish ponds were limed. This is why this bad kind did not occur.*	W	10
52.	6	Oniscidea e.g. *Armadillidium vulgare* woodlice	*pincebogár* (cellar bogár), *krumplibogár* (potato bogár)	*If you touch it, it will become a ball. / Where there is potato and the soil is wetter, it would winter there.*	H	51
53.	7	*Mantis religiosa** European praying mantis	*imádkozó sáska* (praying sáska), *sáska, alázatos manó* (humble imp)	*It’s hands are like if it would pray, but it doesn’t. / They are usually at the watersides. / It becomes rare. Because of the poisons. Mostly it is encountered on grazing land. / We mostly have these green ones, but you could find some brown ones as well.*	A, O	42
54.	7	*Acrida hungarica** Hungarian snouted grasshopper	*sáska*	*It leaps like magic. / They come in different colours.*	G, O	2
55.	7	*Oecanthus pellucens* Italian tree cricket	*őszifíreg* (Autumn worm) *őszike* (little in autumn), *haris* (roarer)	*It says ‘gather, gather’. / Autumn is here, the autumn worm sounds. / It comes out only in the evening. / It was brought in on flower vases on the leaves.*	O	40
56.	7	*Locusta migratoria** migratory locust	*siska, sáska, szöcske* (hopper)	*This does not sing, it grazes. It was here long ago, now is gone. / Hay meadows were stripped barren. We collected them.*	A	11
57.	7	*Tettigonia viridissima* great green bush-cricket	*sáska, kabóca, szöcske* (hopper)	*It grows high. / Flies and jumps as well. / It likes to be in the reeds on sedges, weeds. / Haven’t seen it for a few years. / It likes to eat leaves, comes in the house. Causes panic, although it does not bite your head off.*	O	73
58.	7	*Dociostaurus maroccanus* Moroccan locust	*sáska*	*A bad lot, eats away everything. They fly. / We had them before, in 1951 for the last time.*	O	9
59.	7	*Calliptamus italicus* and similar species Italian locust	*szöcske* (hopper), *sáska*	*They fly.*	G	7
60.	7	*Chorthippus* spp. e.g. *Chorthippus parallelus* smaller grasshoppers	*szöcske (hopper), sáska, kabóca*	*This tiny thing is on the hay meadow. But I don’t know the name. / You get green ones, brown ones.*	G	63
61.	7	*Gryllus campestris* field cricket	*tücsök, mezei tücsök* (meadow tücsök)	*Black cricket. It is lured out of the hole with a blade of grass. / It can not jump any more when you crossed it with your finger. As long as it did not return to the ground. Then it would be able to jump again. / It makes music in summer and does not care with the winter. / It has a kind of wing but light. / They would be in the same hole with the dung-beetle* (*Geotrupes* spp.)*. I would say it is menial of the other one.*	G	95
62.	7	*Acheta domestica* house cricket	*tücsök, házi tücsök* (house cricket), *fehér tücsök* (white cricket)	*Brown-reddish. / Sing in the night. / Well, it leaps, giant leaps. / Long ago it was there in bakeries and in old peasant houses in the door case. / It likes to come in the house, crawls to and fro all winter. / Behind the refrigerators, because it is both warm and damp. / It would loose its colour in the house and sometimes will be quite white by the time it comes out in Spring.*	H	33
63.	6	*Gryllotalpa gryllotalpa* European mole cricket	*lótetű* (horse louse), *lóhere* (horse drone), *csúr, csikóbogár* (foal beetle), *medve* (bear), *ollós bogár* (scissors beetle)	*It can be found in manure. / Eats worms. And the mole eats them and worms alike. / It would make big troubles in seedling beds. / It is called louse, but it’s not so tiny to be a louse. / Flies in the night. / It has millions of tiny eggs in the nest. / Around Losonc it was called a bear. It resembles it.*	S	98
64.	6	*Pyrrhocoris apterus* firebug	*suszterbogár* (cobbler bug), *csiribabó, verőkőtő, kőverő* (stone beater), *bodobács*	*Nice beetles. The first one to come out in Spring to the sun. / ‘They stick together like the csiribabó’* (firebugs)*. / ‘They sit out like the verőkőtő’. / Usually on rotten trees.*	S	100
65.	6	Notonectidae e.g. *Notonecta glauca* backswimmers (true bugs)	*vízibolha* (water flea)	*This kind of bug is in the water, two legs are long. / If the net was any denser, they would eat up smaller fish in the apex. / Jumps and bites.*	W	10
66.	8	*Gerris* spp. e.g. *Gerris paludum* water striders	*vízipók* (water spider), *vízimolnár* (water miller), *vízibizigli* (paddle boat)	*Collects lesser bugs on the surface of the water. Very quick. Always on the top of the water. Maybe it was called water miller for this reason. / We also called it paddle boat. They run in groups. / They can play on the water very well.*	W	58
67.	6	*Dolycoris baccarum* and similar species sloe bug (true bugs)	*büdösbogár* (stink bogár), *büdösbence* (stinking Ben), *poloska, büdösmártin* (stinking Martin), *büdösbanka (*stinky banka), *mezei poloska* (meadow poloska)	*Sometimes you snatch it with raspberries. It’s bitter. And very stinky. / Before cold weather comes, they are already between the window panes. They know winter is coming.*	H, O	98
68.	6	*Palomena prasina, Nezara viridula* green shield bug southern green stink bug	*büdösbogár* (stink bogár), büdösbence (stink Ben), *poloska*	*Do you know, which is green? The one born this year. By next year it will be the same colour. This is like a swan. A young swan is greyish mottled.*	H, O	82
69.	6	*Graphosoma lineatum* Italian striped-bug	*büdösbogár* (stink bogár)	*Lives on dills. Each stem has 10 or 15. / They suck out moisture up at the seeds. / If you only touch any of them, they are stinky.*	S	15
70.	6	*Eurydema ornatum* red cabbage bug	*káposztabogár* (cabbage bogár), *büdösbogár* (stink bogár)	*The same smell as [Dolycoris baccarum]. / Eats cabbage. / Comes in lots.*	S	14
71.	6	Carabidae e.g. *Zabrus tenebrioides* ground beetles	*bogár*	*They are running about. Here in the greenhouse. / When the grave is dug, you would see such black bugs often in the ground.*	H	42
72.	6	*Geotrupes* spp. e.g. *Geotrupes vernalis* dor beetles	*ganajtúró* (dung grouter)	*Grouts in cow dung. Undemanding beast. / Makes pellets and rolls them. / There were millions. Today only now and then.*	S	53
73.	6	*Melasoma populi* poplar leaf beetle	no name	*It would come on poplars in the woods. Crawls on leaves.*	F	4
74.	6	*Phytodecta rufipes* brassy willow leaf beetle	*ötpettyes katicabogár* (five spots Kate bogár), *katicabogár* (Kate bogár)	*It is lighter, yellowish-red, five spots. Gnawed sown Trifolium away. / A pest in parcels under lucerne.*	A	4
75.	6	*Leptinotarsa decemlineata* Colorado potato beetle	*krumplibogár* (potato bogár), *mandalinka, kolorádóbogár* (Colorado bogár), *kórórágó* (stalk gnawer), *pizsamás bogár* (pijama bogár)	*This is what we got from America. / You must put nettle in water and leave it for week. It will become stinky and sprays the plant. / You could get paid if you found such bugs. / It was introduced with the potato. / Just now there are not so many. It rained a lot.*	A	100
76.	6	*Chaetocnema* spp. and *Phyllotrema* spp. e.g. Chaetocnema *tibialis* flea beetles	*balha, káposztabolha* (cabbage bolha)	*It jumps. Makes holes in radish, kohlrabi leaves, cabbage. / Tiny black bugs.*	A	61
77.	6	*Chaetocnema* spp. and *Phyllotrema* spp. (+*Erwinia stewartii*) flea beetles and Stewart’s wilt	*balha, bogár*	*Tiny black bugs, leaves long marks on the greenish part of maize. / Sucks the leaves, likes sweet corn best.*	A	4
78.	6	*Epicometis hirta* hairy beetle	*szőrös kandalló* (hairy hearth), *bogár*	*Hairy. / Comes on flowers. There are many, in particular on the fields, sunflowers and wheat.*	A	11
79.	6	*Tenebrio molitor* mealworm beetle	*drótkukac* (wire worm), *lisztkukac* (flour worm)	*I bought corn meal in the shop and it was full with it. / It also breeds in ground pepper. / You had better screen the flour before use.*	H	14
80.	6	Curculionidae e.g. *Larinus turbinatus* true weevils	*orrúbogár* (nosy bug)	*It has a long trunk. / The wings are hard.*	A	10
81.	6	*Anthonomus pomorum* apple blossom weevil	*bimbólikasztó* (bud puncher)*, bimbólikasztó bogár* (bud puncher bogár),	*A tiny bug, gets into the buds when it starts to sprout and does harm to cherries, plums.*	O	19
82.	6	*Cleonus punctiventris* sugar-beet weevil	*répabogár* (beet bogár)	*Carrot beetle. Gnaws a hole in the carrot. Sucks the sap of tiny carrots and they perish, wither. This is why it was controlled by spraying.*	A	2
83.	6	*Ceuthorrhynchus macula-alba* poppy ceutorrhynchid *beetle*	*mákbogár* (poppy bogár)	*Punches poppy heads while young. And it would not yield because the worms eat it away from inside.*	A	2
84.	6	*Sitophilus granarius* wheat weevil	*zsúzsok, búzazsúzsok* (wheat zsúzsok)	*It is also a bad lot, eats the wheat. / Does harm to fodder inside. / If there is only a little water, it would grow.*	H	22
85.	6	*Bruchus pisi, Acanthoscelides obtectus* pea beetle, bean beetle	*zsúzsok, paszulyzsúzsok* (bean zsúzsok)	*Comes from inside the peas. / More recently you can find in beans as well. / All beans must be discarded. Eats the cotyledon out.*	H	58
86.	6	*Lytta vesicatoria* Spanish fly	*kőrisbogár* (ashtree bogár)	*My grandmother would say, rain is coming the ash tree stinks. / Very stinky, in particular on the ash tree.*	F, G	88
87.	6	Cantharidae e.g. *Rhagonycha fulva* soldier beetles	*kőrisbogár* (ashtree bogár)	-	F, G	7
88.	6, 10	*Melolontha melolontha* cockchafer	*cserebogár, májusi cserebogár* (May cserebogár)*, pajod, csimmaz, pillangó* (butterfly)	*You can get cockchafer in Spring. Not later. / You need three years before it develops. / If there is a lot of cockchafers, you will get high corn yields. / Cockchafer would lay* (give birth to) *that white butterfly.*	O	100
89.	6	*Amphimallon solstitialis* summer chafer	*cserebogár, kis cserebogár* (small cserebogár)	*It comes later on, in June, mostly* (as opposed to the ordinary cockchafer)*. / This is lesser and yellowish.*	O	5
90.	6	*Cerambyx cerdo* great capricorn beetle***	*cincér, hőscincér* (hero cincér)	*It can weep like hell when you get it. / Got large moustache and long legs. / ‘Your moustache is like that of a capricorn beetle.’*	S, F	33
91.	6	*Rosalia alpina** Rosalia longicorn	*cincér*	-	F	2
92.	6	*Pyrrhidium sanguineum* Welsh oak longhorn beetle	*kőrisbogár* (ashtree bogár)	-	S	4
93.	6	*Lucanus cervus** stag beetle	*szarvasbogár* (horn bogár), *csikkantós bogár* (pinching bogár)*, bika, tehén* (bull, cow)	*Once you catch it, it would grasp your finger like a pair of scissors. / We would nail it on the wall, the kids just gazed. / This is the bull* (male)*, and this is the cow* (female)*. / They wrestle. Two bulls. / It comes mostly around oak trees.*	F	98
94.	6	*Oryctes nasicornis** European rhinoceros beetle	*orrszarvúbogár* (nose horn bogár), *orrszarvú* (nose horn), *szarvasbogár* (horn bogár)	*You can’t get it everywhere. / They like old trees. / A kind of horn beetle.*	F, S	61
95.	6	*Lethrus apterus** flightless earth-boring dung *beetle*	*bogár*	*This bores holes in the ground and drags in leaves in reserve gear.*	G	2
96.	6	*Pentodon idiota* beetle species	*bogár*	*This is in springtime. Those big ones on the sidewalk.*	S	4
97.	6	*Coccinella septempunctata* seven-spot ladybird	*katicabogár* (Kate bogár)*, hétpettyes katicabogár* (seven spots Kate bogár), *petikebogár* (Pete bogár)	*It eats aphids. It seems it likes them. / ‘Kate beetle! Where I go to marry?’ And then we watched. Blew at it till it flew away. That way we married.*	S, G	100
98.	6	*Adalia bipunctata* two-spot ladybird	*katicabogár* (Kate bogár), *petikebogárka* (little Pete bogár)	*Similar to Kate beetle, but has only two spots, unfortunately.*	S, G	12
99.	6	*Psyllobora vigintiduopunctata* 22-spot ladybird	*katicabogár* (Kate bogár), *sárga katicabogár* (yellow Kate bogár), *11 pettyű katica* (11 spots Kate)	*There are yellow Kate beetles as well.*	S, G	68
100.	6	*Harmonia axyridis* harlequin ladybird	*katicabogár* (Kate bogár)	*There was such a Kate beetle invasion last year. They are not the ones I saw when I was a kid. / The Sun shone there in a warm afternoon, there were so many you could grasp them.*	H	71
101.	6	*Meloe* spp. e.g. *Meloe proscarabaeus* (*) oil beetles	*bogár*	*It’s got a big belly like this.*	F, A	11
102.	6	*Calosoma sycophanta** forest caterpillar hunter	*bogár*	*Usually it is on the ground as well. When we get home, it sneezes. You are not hit by what it blows out. Maybe only a little air. Protects itself. / Runs away quickly.*	F	7
103.	6	Gyrinidae e.g. *Gyrinus natator* whirligig beetles	*bogár*	*-*	W	4
104.	6	*Dytiscus* spp. e.g. *Dytiscus marginalis* great diver species	*vízibogár* (water bogár)	*-*	W	4
105.	6	*Hydrous piceus* great silver water beetle	*vízibogár* (water bogár), *csíbor* (pincher), *vízibölény* (water buffalo)	*Big and black, likes warm water. / Mostly in lakes. / Sets on the fish, gnaws on it. / They say it bites. / When it ebbed, they flew here to the light.*	W	79
106.	6	*Cetonia aurata* green rose chafer	*szentjánosbogár* (Saint John’s bogár), *foszforbogár* (phosphorus bogár), *aranyos virágbogár* (golden flower bogár)	*All say glow worm because it shines as the Sun hits it. But this can not be seen in the night. / Flies with a buzz. Creeps into drying cloth.*	G	82
107.	6	*Lampyris noctiluca, Lamprohiza splendidula* common glow-worm, Central European firefly	*szentjánosbogár* (Saint John’s bogár), *foszforbogár* (phosphorus bogár)	*They are tiny and light up. You can see in the night air. / It’s only a small worm. I would illuminate when the [Oecanthus pellucens] sounds. The back is lighting.*	G	69
108.	6	*Ips* spp. e.g. *Ips typographus* engraver beetles	*szu, szujétel, faszu* (wood szu)	*It makes very small holes, tiny dense holes. / I would put firewood in tin trays to keep it from the parquet.*	H	100
109.	6	*Agriotes* spp. e.g. *Agriotes sputator* click beetles	*drótkukac* (wire kukac), *drótbogár* (wire bogár), *drótféreg* (wire féreg)	*Likes carrots. Yellowish, hardy.*	H	27
110.	6	*Diabrotica virgifera* Western corn rootworm	*kukoricabogár* (corn bogár)	*Can make a lot of harm. Sucks the sap of the leaves.*	A	2
111.	6	*Blaps* spp. e.g. *Blaps lethifera* tenebrionid *beetle*	*büdösbogár* (stink bogár), *büdösbanka* (stinky banka), *kátránybogár* (tar bogár), *svábbogár* (Swabian bogár)	*Very stinky when you step on it. / Mostly in cellars. Formerly tar paper was put down in dirt floored houses and it was underneath.*	H	33
112.	4	*Xylocopa violacea, Xylocopa valga* black colored carpenter bees	*dongó* (buzzer), *cigánydongó* (Gypsy buzzer)	*Big, black and collects honey also. Bites. Has a loud buzz. / Does not bite. / I’ve got a barn full of wood. They drill on them like drilling machines. It laid eggs into it.*	S	53
113.	4	*Apis mellifera* European honey bee	*méh, méhecske* (little méh), *házi méh* (house méh)	*It was August when there is less flowers and these times bees are more dangerous, attack you easier.*	O	100
114.	4	*Apis mellifera* var. *ligustica* Italian bee	*vadméh* (wild méh), *erdei méh* (forest méh)	*It was an old house, full of wild bees in the end. They sting, its painful. / We call them forest bee. Once we brought some home with my father. Ants attacked and killed them off. / They make hives in hollows. / The tree brings that wax. And they eat it.*	G, F	31
115.	4	*Osmia adunca* mason bee species	*nádiméh* (reed méh)	*It can nest in a single stem of reed, but can go up to the first node only. / They do not bring in honey, but pollens, and they put a lid on it made of mud, which is gnawed away by the young when they come out of it.*	H	18
116.	4	Andrenidae e.g. *Andrena flavipes* mining bees	*csemelyke, vadméhe* (wild méh)	*It is in the ground. Such brownish in colour. Collects nectar, pollen. Tiny. / Leaves little heaps around with a hole in the middle. It does not have a strong sting but it does sting. It’s got a net but not so beautiful which is built by the common wasp.*	G	15
117.	4	*Bombus terrestris* buff-tailed bumblebee	*dongó* (buzzer)*, csemélyke, földiméh* (ground méh)	*Hairy. They dwell in the ground. Don’t do any harm to man. Collect honey as well. / Maybe there is a yellowish stripe in the back on top. / Like my finger – they make little round nests to put honey in it for themselves. Foxes, dogs, mice rake it out and eat.*	G, O	25
118.	4	*Bombus lapidarius* red-tailed bumblebee	*dongó* (buzzer)	*It does not die as the other bee which has the sting coming out. This one does not tear its sting off, it merely stings you. / Such a big black with red and yellow head.*	G, O	7
119.	11	*Formica rufa** red wood ant	*vöröshangya* (red hangya), *erdei vöröshangya* (forest red hangya)	*Where there is this big ant, it makes a big ant hill. If you poke it millions come out, but they are all alike.*	F	14
120.	11	ant casts with wings	*szárnyashangya* (winged hangya)	*It drills the ground and then billions fly around in the air. / It comes out from the parquet in the apartment. / We would say if the winged ant comes out, it will rain.*	S	85
121.	11	*Camponotus* spp. e.g. *Camponotus ligniperda* carpenter ants	*tököshangya* (ballsy hangya), *hangya, fekete hangya* (black hangya)	*They gnaw at trees. / We could not stay there under the walnut tree* (sitting, talking) *there were so many. I put salt on their way and later I could see that they move away. Now see, that one came back, just like talking something to the others and they queue up and move out. I say, cunning beasts, they are. That one was the officer, who commanded. / It’s ballsy because it carries the eggs between the legs like a big squash. / I brought into the flat with the wood.*	F	42
122.	11	*Lasius flavus, L. umbratus* yellow meadow ant	*sárga hangya* (yellow hangya)	*That one is yellow, bites like hell. / Like the poppy seeds, so tiny they are.*	A	21
123.	11	*Temnothorax* spp. e.g. *Temnothorax affinis* ant species	*hangya*	*Galls grow on oak trees. Some kind of tiny ants are fond of living in them. They make a little hole in it where they can get through. They would thrive on the gall.*	F	2
124.	11	*Tetramorium caespitum* and similar ant species	*hangya, fekete hangya* (black hangya)	*There are so many in the garden it is like a miracle. They climb on our legs but do not bite so hard. Tiny black bits.*	S	100
125.	4	*Sceliphron destillatorium* mud dauber wasp	*darázs, szalmadarázs* (straw darázs)	*Makes nest of mud on the rafter. / It’s long and thin.*	S	20
126.	4	Sphecidae e.g. *Ammophila sabulosa* digger wasps	*földidarázs* (ground darázs)	*Drills a hole in the sand. Come and go in it.*	G	2
127.	4	*Vespa crabro* European hornet	*lódarázs* (horse darázs)	*That one is dangerous. Some 5–6 bite you, you may die. / We say, if nine horse wasps bite the horse, it will perish. / It made a 12 storey nest, hanging on the wall. / You had to pee on the ground and smear the mud over it* (the bite)*. / Many are allergic when bitten. It would not die after biting. Goes on, bites again.*	F	100
128.	4	*Vespula vulgaris, Polistes gallicus* paper wasp species	*kecskedarázs* (goat darázs), *darázs, házi darázs* (house darázs)*, padlási darázs* (attic darázs)	*My whole head was swollen. I was a kid, the old women gathered around, some brought sour cream, this or that. I was smeared over, embalmed. Next day I was okay. / Likes mainly the attic. / The mother survives in winter and then there are many in summer. / Aphid appear on the young upper leaves of the peach tree, then the wasp comes but they do not do any harm to the lice I think they eat what the aphid produce.*	S	61
129.	4	*Paravespula germanica* German wasp	*darázs, földidarázs* (ground darázs)	*They put fire above the nest, they were poured over with hot water, put chemicals on it. / It had a tiny hole like this. They would keep on coming and going there and built a beautiful honeycomb.*	S	100
130.	15	*Cynips quercusfolii* gall wasp species	*cseregolyó* (oak ball)*, gubacs*	*This is not the fruit of the tree, it is on the leaves. On oak trees. / There is some bug in this ball as well. Hatches from it.*	F	37
131.	15	*Rhodites rosae* mossy *rose* gall *wasp*	*gubics, hamufíreg* (ash worm), *macskatöki* (cat’s bollocks)*, csipkelabda* (rose hips ball)	*It’s on briars. I saw it on roses. Good for nothing like the balls of a cat.*	G	11
132.	15	*Andricus hungaricus* Hungarian gall wasp	*gubacs* (gall), *gubics* (gall)	*My mother made us thrown them away. You must not keep it* (at the house) *because brood will not hatch the eggs.*	F	83
133.	14	*Acherontia atropos** Death’s-head hawk moth	*ördögpillangó* (devil’s pillangó), *boszorkánylepke* (witch lepke), *halállepke* (death lepke), *halálfejes lepke* (death’s head lepke)	*Quite big. A pest. / Big and ugly like the devil. / They say it was a witch butterfly, but this is wrong. Some old bitches made it up. / Comes in the evening and flies around here. / It has a big death's head.*	S	77
134.	14	*Macroglossum stellatarum* hummingbird hawk-moth	*lepke, kocsisirma* (carman Irma), *kolibri* (hummingbird)	*Has a long tongue reaching in every flower. / Is like a hummingbird. / Make noises with the wings.*	S	36
135.	14	Lepidoptera e.g. *Melitaea athalia* butterflies	*lepke, pillangó, hernyó*	*Not a real pest. We were glad to see them long ago. They are very nice.*	G	100
136.	14	*Ephestia kuehniella* Mediterranean flour moth	*moly*	*A little worm. When you screen the flour, it’s like a spider web, woven in. Flour gets the moth when you keep it long, let’s say a year.*	H	7
137.	10	*Coccyx turionella* pine bud moth	*rügyfúró* (bud driller)	*They can make big damage when they are many. Does harm to cherry trees in springtime mostly. This is long like this, has a bill, it punches the plants with.*	O	2
138.	10	*Cydia pomonella, Anarsia lineatella* codling moth, peach twig borer	*kukac, hernyó*	*Moth larvae are called worms here. Likes to take a place in quince.*	O	9
139.	14	*Inachis io** European peacock butterfly	*pávaszemes lepke* (peackock lepke), *pávaszem* (peackock’s eye)	*If you see a red one first in the new year, you will remain healthy and fall in love.*	G	9
140.	14	*Vanessa atalanta** red admiral	*lepke*	*-*	O	2
141.	14	*Vanessa cardui* painted lady	*lepke*	*It was like this which came from Africa* (heard on the radio)*. And next year I saw two or three but never again. Maybe it would not find food to stay.*	O	2
142.	14	*Gonepteryx rhamni* common brimstone	*sárga lepke* (yellow lepke), *sárga pille*	*If you see a yellow butterfly first in spring, you’ll fall ill.*	G	7
143.	14	*Pieris brassicae* large white	*káposztalepke* (cabbage lepke), *lepke, fehér lepke* (white lepke)	*Lays eggs on the cabbage and a little green worm comes out of it. / Its wings are white and there are plenty. Cockchafer*s l*ay its worm. You say when there are many cockchafer*s*, there is plenty of worms on the trees.*	A	86
144.	14	*Polyommatus* spp. e.g. *Polyommatus icarus* blues (butterfly species)	*lepke, pillangó, kék pillangó* (blue pillangó)	*After the rain these white butterflies are in the edges of puddles. As many can get to it.* / *Bluish grey. It was small.*	G	31
145.	14	*Mamestra brassicae* cabbage moth	*bagolylepke* (owl lepke), *éjjeli pille* (nocturnal pille)	*Eats cabbage, cauliflowers, broccoli. When you see plenty of butterflies, soon the worms will come. / It comes through the window when it is open.*	A	11
146.	14	*Tineola biselliella* common clothes moth	*moly, molylepke*	*Eats your clothing. / The little moth worms.*	H	96
147.	14	*Ostrinia nubilalis* European corn borer	*málépillangó* (maize pillangó), *molylepke*	*If you are careless and leave old corn in the silo and put the new one on top, that one will be two years old and definitely infested with it. / Doesn’t eat the corn, just the germ. / When corn is over, half a handful of dust is left. It is butterfly droppings.*	S	70
148.	14	*Catocala elocata* French red underwing	*bagolylepke* (owl lepke)	*The one with the red back wings.*	A	2
149.	14	*Helicoverpa armigera* cotton bollworm	*bagolylepke* (owl lepke)*, paszuly-molypillangó* (beans molypillangó)	*It goes into the geranium buds and eats them from inside. / Likes to attach to trees. And to the walls, frequently comes in. / When it comes out of the bean, a hole is left behind. A little white worm.*	A	23
150.	14	*Cossus cossus* goat moth	*szúlepke* (engraver beetle lepke)	*Big brown butterfly. Puts eggs into the bark. It gets into timber laid a long time raw with the bark on. They are almost as big as a grub. Gnaw out passages like a pencil.*	S	5
151.	14	*Saturnia pyri** giant peacock moth	*boszorkánypille* (witch pille), *halállepke* (death lepke), *szemeslepke* (eyed lepke)	*They are big, appear late in the evening when you put the lights on. / Superstitious folks would nail it above the door.*	O	16
152.	14	*Iphiclides podalirius** scarce swallowtail	*fecskefarkú lepke* (swallow tail lepke), *fecskeszárnyú pillangó* (swallow winged pillangó)	*The same* (as *Papilio machaon*)*, only with a different colour and patterns. / The wings have forked ends.*	G	14
153.	14	*Papilio machaon** common swallowtail	*fecskefarkú lepke* (swallow tail lepke), *fecskeszárnyú pillangó* (swallow winged pillangó)	See above	G	14
154.	14	*Lymantria dispar* Gypsy moth	*gyapjaspillangó* (woolly pillangó), *gyapjaslepke* (woolly lepke)	*Worm, eats away the leaves of a tree. One year it grazed off all leaves by the first of July. Within less than one and half months it was green again because what nature can spoil, can make it right again.*	F	56
155.	14	*Bombyx mori* domesticated silkmoth	*selyemhernyó* (silk hernyó)*, selyemlepke* (silk lepke)	*They had to go each day, picked the mulberry tree leaves for them.*	H	4
156.	15	*Aedes* spp. e.g. *Aedes vexans* mosquito species	*szúnyog, baglinc*	*Can cause inconvenience. / Has the impertinence to enter the house. / Little, thin, but if sucks itself full of blood, the bite would itch, you can’t help it. / When it's rainy, there is plenty, and evenings in marshy places. / I got the malaria once from a mosquito bite. I was a little girl.*	H	100
157.	6	Ceratopogonidae e.g. *Culicoides imicola* biting midges	*báglinc, muslinca*	*Tiny beasts, like a bigger poppy seed. / It was black, but it is also bad. / There was plenty on the meadow in floods. / Harder and smaller than the mosquito. / If you smear it over with vinegar, itching will go away.*	F	14
158.	14	Psychodidae e.g. *Clogmia albipunctata* moth flies	no name	*There is a million in the bath. Both summer and winter.*	H	13
159.	6	*Drosophila* spp. e.g. *Drosophila melanogaster* fruit flies	*muslica, muska, baglinc*	*There are millions. When the wine works. / Doesn’t go into the must.*	H	100
160.	15	*Tipula* spp. e.g. *Tipula maxima* crane flies	*szúnyog, szúnyogkirály* (king szúnyog), *árvaszúnyog* (orphan szúnyog), *apatini szúnyog, óriásszúnyog* (giant szúnyog)	*It does not bite, only flies around in the house. / The long legged one does not creep on you. / We stroke the mosquito king to death, there will be no mosquitoes now – this is how we said.*	H	60
161.	5	*Tabanus bovinus* pale giant horse-fly	*bögöly, pécsik*	*It’s got streaked eyes. / Bites you as well, but cows get them in lots! / When you sweat, it will attack you.*	P	11
162.	5	*Oestrus ovis* sheep bot fly	*bogár*	*A fly lays eggs in the nostril of the sheep. When developed, the sheep would blow it out. We would always see two or three in the fodder trough.*	P	4
163.	5	*Gastrophilus intestinatus* horse bot fly	*lóbögöly* (horse bögöly)	*-*	P	2
164.	5	*Haematopota* spp. e.g. *Haematopota pluvialis* clegs (horsefly species)	*pécsik, pécsiklégy, bögöly, lólégy* (horse légy), *bogaraztató légy* (make-jump légy)	*It bites the cows in summer. Horses even more. Greyish. Bite more before rain. / From beginning, mid-July up to mid-August is the season when they attack livestock in big numbers.*	P	83
165.	5	*Hypoderma bovis* warble fly	*iméj, böge, dongólégy* (buzzing légy), *zigarzóbogár*	*Winters under the skin of wild game. Long ago they were there under the skin of the cattle, livestock. / We would press them out from the back of the cows. / When it started to buzz, it rounded up livestock like a dog.*	P	77
166.	5	*Lucilia* spp. e.g. *Lucilia caesar* blow flies	*döglégy* (carcass légy), *beköpőlégy* (spiting légy)	*Shiny, mostly on droppings. / You must not have meat left exposed because flies would have spat on it.*	S	73
167.	5	*Calliphora vicina* bluebottle blowfly	*döglégy* (carcass légy), *köpőlégy* (spiting légy)	*Spit on meat* (=lays eggs on meat)*. / Bluish ones are bigger than the green ones.*	S	21
168.	5	*Fannia canicularis* lesser house fly	*légy, kutyalégy* (dog légy), *istállólégy* (stable légy)	*Little black. Not so noisy like an ordinary fly. And keeps on flying around the lamp.*	H	15
169.	5	*Musca domestica* housefly	*légy, házi légy* (house légy), *pusztuljka* (little perish)	*It’s not so dangerous which is in the house. / When flies bite, it will rain, they say.*	H	100
170.	5	*Stomoxys calcitrans* stable fly	*légy*	*Smaller with a pointed nose. They would bite my leg.*	S	2
171.	5	*Haematobia irritans* horn fly	*óli légy* (pen légy), *légy*	*That one is longer nosed, but smaller, bites so hard that you jump.*	S	7
172.	5	*Sarcophaga carnaria* common flesh fly	*nyűszaró* (maggot shit), *dongólégy* (buzzing légy), *köpőlégy* (spiting légy)	*Big blacks also spat on the meat. And then the maggots gnawed out the meat. / Big and humming, flies quickly. / Maybe this lays most because it is directly full with those tiny worms. You can see it when you squash it.*	S	66
173.	5	*Simulium* spp. e.g. *Simulium colombaschense* black flies	*kolombácsi* (originated from Kolumbács)	*This attacks the livestock, cows intensively. They say, it may happen that the cow will perish when bitten. / This fly was here in 1938 and it was drummed out that everybody should take care and not go to the meadows too frequently, because you will get bitten. / We put* (dry) *manure in a pot and walked around the grandma so she could hoe and this fly did not hurt her. We made smoke of it.*	S	25
174.	9	*Melophagus ovinus* sheep ked	*juhkullancs* (sheep kullancs)*, kullancs*	*Stays in sheep. Flat. / Gnawed the wool.*	P	4
175.	5	*Hippobosca longipennis* dog fly	*kutyalégy* (dog légy)	*Sticks on the dog, you can hardly get rid of it.*	P	2
176.	10	*Rhagoletis cerasi* s.l. cherry fruit fly	*kukac*	*My mother would say we should not look for it, because the tiny worm was created in it. Well, it was, because it was put in it during blossoming.*	O	94
177.	10	*Rhagoletis pomonella* apple maggot	*almalégy* (apple légy)	*In fact not all are the same, this one gnaws at the apples only.*	O	10
178.	10	*Psila rosae* carrot fly	*fíreg, murokfíreg* (carrot worm)	*Little soft white worm.*	S	6
179.	10	*Delia radicum* cabbage fly	*káposztaféreg* (cabbage féreg)	*Gnaws on cabbage leaves, but what it will become, we do not know.*	A	12
180.	9	*Braula coeca* bee louse	*méhkullancs* (bee kullancs)	*A tiny animal. / You get them on bees, this red one. It kills the bees. / When you neglect their management, it’s when it comes.*	P	6
181.	9	Aphididae (green) e.g. *Aphis pomi* green colored aphid species	*levéltetű* (leaf tetű), *tetű, ződtetyű* (green tetű)	*That one is green. It does not hatch* (does not lay eggs)*, it litters* (gives birth)*. The ladybird eats them. The dropping is sweet. Ants would climb the tree to feed on it. / It is under the leaves. It sucks and the leaf goes dry. If there are many on the peach tree, wasps would also gather.*	O	100
182.	9	*Myzus cerasi* black cherry aphid	*fekete levéltetű* (black leaf tetű), *levéltetű* (leaftetű)	*The black leaf louse likes cherries better. / The edges of the leaf curl up because it sucks on it.*	O	16
183.	9	*Dysaphis plantaginea* rosy apple aphid	*szürketetű* (grey tetű)*, tetű*	*Tiny and grey. Water with bordeaux mixture, it will be killed off. A little weak something. The leaf would curl up.*	O	21
184.	9	*Planococcus citri* citrus mealybug	*gyapjastetű* (woolly tetű)	*This one is like if it was mouldy. It is mostly at the stem of leaves. This does not fly, where does it generate, where does it come from, I do not know.*	O	4
185.	9	Aleyrodina e.g. *Aleyrodes proletella* whiteflies	*hamufíreg* (ash fíreg), *tetű, pillangó*	*The twig would go entirely white. / Those whites are the butterflies. It does harm to the grapevine. Gnaws at it when in blossom.*	A	20
186.	9	*Pulex irritans* human flea	*bolha*	*Jumps, tiny and black. / We would put walnut tree leaves in the neck to the shirt, so they did not hurt us.*	P	100
187.	9	*Ctenocephalides canis* dog flea	*bolha, rókabolha* (fox bolha)	*There are many fleas in the dog which also attacks man. / That one would bite you as well, but it does not stay, does not like it so much.*	P	6
188.	9	*Viteus vitifolii* vine louse	*filoxera*	*-*	O	2
189.	9	Coccoidea e.g. *Quadraspidiotus perniciosus* scale insect	*pajzstetű* (shield tetű)	*We did not have it when I was a child. / It’s like an armour on the back. Eats away the leaves but the stems even more. / That one is here on the plum tree. Sometimes it’s not any more only the house. It has left it.*	O	7
190.	9	*Eriosoma lanigerum* woolly apple aphid	*vértetű* (blood tetű)	*-*	O	4
191.	9	*Pediculus humanus capitis* head louse	*tetű, fejtetű* (head tetű)*, hajtetű* (hair tetű), *serke*	*Climbs on you, on your head. / It’s not a shame to get it, only to keep it. / It was healed at the house. We mixed oil, spirit and petrol and smeared with it. We still have it. It’s because untidiness.*	P	100
192.	9	*Menacanthus stramineus* chicken body louse	*tyúktetű* (hen tetű), *tetű*	*It does not stick to man. / It was controlled by onion oil. You must roast onions and smear it. / Pig fat, small red peppers were put in it, and [Sambucus ebulus] in the pen.*	P	91
193.	9	*Haematopinus suis* hog louse	*tetű, disznótetű* (pig tetű), *sörte*	*It’s greater than the other lice, but does not live of man.*	P	29
194.	9	*Bovicola bovis* red louse	*tetyű*	*Cattle would be smeared over with tobacco juice. There is none any more.*	P	20
195.	9	*Pediculus humanus humanus* body louse	*ruhatetű* (cloth tetű), *tetű*	*Which lives in the cloth, would not go on your head. / That one is white.*	P	91
196.	9	*Pthirus pubis* crab louse	*lapostetű* (flat tetű)	*They say it’s on your loin only. Nowhere else.*	P	8
197.	9	*Cimex lectularius* bed bug	*poloska*	*You had to sleep with the lights on because in the dark the plant bug would come out. / They occurred only during the war.*	P	27
198.	6	*Blatta orientalis* oriental cockroach	*csótány, svábogár* (Swabian bogár)	*In the blocks of flats. Totally black beetle, a larger one. / They creep under the cupboards. / If you put on the light, it will disappear.*	H	23
199.	6	*Blattella germanica* German cockroach	*csótány, svábogár* (Swabian bogár)	*They like to stay in neglected, abandoned kitchens.*	H	12
200.	6	Dermaptera e.g. *Forficula auricularia* earwigs	*fülbemászó* (ear crawler)	*Climbs in your ear and drills it. A bad lot. It has a little dart. / Some went deaf. / Runs away quickly, thin and long. / Cloth, coat was left hung on the tree, this would enter there.*	O	92
201.	7	Cicadinae (except *Cicada orni*) e.g. *Cicadella viridis* cicadas	*sáska, szöcske* (hopper)	*A little bouncing bug.*	G	7
202.	7	*Cicada orni** bigger cicada species	*őszike* (little autumn), *trücsök*	*It is able to sound for hours* (vocalisation)*. Starts to buzz at harvest time. It is able to howl for 10 minutes, for 20 minutes the same tone. / It’s rare in our region, but there are many of them at the sea* (Adriatic)*.*	O	7
203.	15	Cercopidae e.g. *Philaenus spumarius* froghoppers (cicad species)	*hab* (foam)	*It might be a kind of saliva. / There are little ash worms in it, that’s why.*	G	74
204.	6	Myrmeleontidae e.g. *Myrmeleon formicarius* antlions	*porvaatka* (dust mite), *bogár*	*There is a lot of dust under the barn, where it is dry, it makes that nest. But it turns around with such a speed it would scatter dust on both sides. / Sparrows pick them out all.*	S	12
205.	14	*Chrysopa* spp. e.g. *Chrysopa perla* lacewings	*szitakötő* (sieve weaver)*, aranyszemű fátyolka* (gold eyed veil)*, lepke*	*Flutters its wings. / Comes in to the light. / Very stinky when you catch it. / Its wings are like a sieve. There is plenty of them in Fall.*	H	29
206.	15	*Palingenia longicauda** Tisa mayfly	*tiszavirág* (Tisza flower), *kérész*	*Short lived. One day and it’s over.*	W	18
207.	15	Odonata e.g. *Sympetrum sanguineum* red dragonflies	*szitakötő* (sieve weaver), *vízipillangó* (water pillangó), *víziszita* (water sieve)	*It’s got a wing like a sieve. / Flies above the water and feeds on mosquitoes. Flies quickly, stops suddenly and hovers.*	W	21
208.	15	Odonata e.g. *Anax imperator* blue dragonflies	*szitakötő* (sieve weaver), *vízipillangó* (water pillangó), *víziszita* (water sieve)	See above.	W	100

Latin equivalent, English scientific name and Hungarian vernacular names of 208 invertebrate taxa with a typical citation for each, main habitat and proportion of informants who knew the taxon. Key places of encounter and habitats are as follows: aquatic, riparian habitat (W); forest (F); grassy, shrubby areas (G); cropland (A); vineyard, orchard (O); surrounding of the house, village, garden (S); on animal/human (P); in the house (H), everywhere (O)
